# Glucocorticoid deficiency causes transcriptional and post-transcriptional reprogramming of glutamine metabolism

**DOI:** 10.1016/j.ebiom.2018.09.024

**Published:** 2018-09-26

**Authors:** Meltem Weger, Benjamin D. Weger, Benjamin Görling, Gernot Poschet, Melek Yildiz, Rüdiger Hell, Burkhard Luy, Teoman Akcay, Tülay Güran, Thomas Dickmeis, Ferenc Müller, Nils Krone

**Affiliations:** a Institute of Metabolism and Systems Research, College of Medical and Dental Sciences, University of Birmingham, Birmingham B15 2TT, UK; b Institute for Organic Chemistry and Institute for Biological Interfaces 4 – Magnetic Resonance, Karlsruhe Institute of Technology, Hermann-von-Helmholtz-Platz 1, 76344 Eggenstein-Leopoldshafen, Germany; c Centre for Organismal Studies (COS), Heidelberg University, 69120 Heidelberg, Germany; d Kanuni Sultan Süleyman Education and Research Hospital, Küçükçekmece, Istanbul, Turkey; e Istinye University Gaziosmanpasa Medical Park Hospital Gaziosmanpasa, Istanbul, Turkey; f Marmara University, Department of Pediatric Endocrinology and Diabetes, Pendik, Istanbul, Turkey; g Institute of Toxicology and Genetics, Karlsruhe Institute of Technology, Hermann-von-Helmholtz-Platz 1, 76344 Eggenstein-Leopoldshafen, Germany; h Institute of Cancer and Genomic Sciences, College of Medical and Dental Sciences, University of Birmingham, Birmingham B15 2TT, UK.; i Department of Oncology & Metabolism, University of Sheffield, Sheffield S10 2TH, UK; j Department of Biomedical Science, The Bateson Centre, Firth Court, Western Bank, Sheffield S10 2TN, UK.

**Keywords:** Zebrafish, Ferredoxin, Adrenal insufficiency, Oxidative stress, Purine metabolism

## Abstract

**Background:**

Deficient glucocorticoid biosynthesis leading to adrenal insufficiency is life-threatening and is associated with significant co-morbidities. The affected pathways underlying the pathophysiology of co-morbidities due to glucocorticoid deficiency remain poorly understood and require further investigation.

**Methods:**

To explore the pathophysiological processes related to glucocorticoid deficiency, we have performed global transcriptional, post-transcriptional and metabolic profiling of a cortisol-deficient zebrafish mutant with a disrupted ferredoxin (*fdx1b*) system.

**Findings:**

*fdx1b*^*−/−*^ mutants show pervasive reprogramming of metabolism, in particular of glutamine-dependent pathways such as glutathione metabolism, and exhibit changes of oxidative stress markers. The glucocorticoid-dependent post-transcriptional regulation of key enzymes involved in *de novo* purine synthesis was also affected in this mutant. Moreover, *fdx1b*^*−/−*^ mutants exhibit crucial features of primary adrenal insufficiency, and mirror metabolic changes detected in primary adrenal insufficiency patients.

**Interpretation:**

Our study provides a detailed map of metabolic changes induced by glucocorticoid deficiency as a consequence of a disrupted ferredoxin system in an animal model of adrenal insufficiency. This improved pathophysiological understanding of global glucocorticoid deficiency informs on more targeted translational studies in humans suffering from conditions associated with glucocorticoid deficiency.

**Fund:**

Marie Curie Intra-European Fellowships for Career Development, HGF-programme BIFTM, Deutsche Forschungsgemeinschaft, BBSRC.

**Research in context section**

Impaired glucocorticoid biosynthesis severely impacts on human health. The pathophysiological mechanisms of altered metabolism due to glucocorticoid deficiency are not precisely understood and warrant further investigation. Such an endeavor is, however, almost impossible in humans. Here, we employed a zebrafish mutant with impaired mitochondrial glucocorticoid biosynthesis to explore global changes in metabolites and gene expression at both the transcriptional and post-transcriptional level. We have defined glucocorticoid-dependent changes in several metabolic pathways, including glutamine metabolism and linked pathways such as glutathione metabolism and *de novo* purine synthesis. Our study will help to focus clinical studies in rare human conditions associated with glucocorticoid deficiency.

Alt-text: Unlabelled Box

## 1 Introduction

Glucocorticoids (GCs) are crucial regulators of important physiological functions including metabolism [45]. Key steps in GC biosynthesis require mitochondrial cytochrome P450 (CYP) type 1 enzymes that are dependent on NADPH-derived electrons to catalyse their oxidative reactions [58,59]. Mitochondrial electron transfer during GC biosynthesis crucially relies on the iron-sulfur (Fe/S) protein ferredoxin [adrenodoxin, (FDX1)] [58,59].

Mutations in steroidogenic enzymes involved in GC biosynthesis cause a variety of inborn conditions in humans with associated pathophysiology [57,58]. Individuals with impaired GC biosynthesis have either a primary adrenal defect or suffer from secondary adrenal insufficiency due to a problem in the pituitary gland. Both types of adrenal insufficiencies are linked to impaired health status including increased mortality and morbidity as well as reduced quality of life in patients [5,21,57]. Current GC replacement regimens in patients struggle to replace GCs in a physiological manner and do not completely restore the health status [36]. This might be part of the observed health problems in patients with adrenal insufficiency. However, only limited information is available on global metabolic changes and pathway dysregulation in GC deficiency, which are likely to play a significant role for the observed pathophysiology and associated co-morbidity in humans. Importantly, conditions associated with isolated GC deficiency in humans are very rare. Therefore, research into the physiological role of GCs and associated disorders greatly benefits from animal *in vivo* models allowing for comprehensive tissue sampling or application of transgenic techniques unfeasible in humans.

The zebrafish (*Danio rerio*) is a well-established vertebrate model for understanding gene function in embryonic development, disease, and metabolism [35,50,67,68], including research on the endocrine system and stress [53]. Similar to humans, zebrafish are active during the day and use cortisol as their main GC [53]. A fully functional stress axis leading to the release of cortisol by the interrenal gland, the fish counterpart to the mammalian adrenal gland [53], is present at four to five days of development [32,38,76]. Several zebrafish mutants [19,23,33,60] and transgenic reporter lines [6,8,24,[28], [29], [30],^37^,^44^,^63^,^75^,^76^] of steroid hormone synthesis and action have been established. They allow for comprehensive analysis of *in vivo* processes and for high-throughput compound screenings to identify novel drug targets. Zebrafish possess two paralogues of the human *FDX* gene, *fdx1* and *fdx1b*, with *fdx1b* being specifically expressed in steroidogenic tissues [33,77] and serving as the only relevant electron donor for mitochondrial steroidogenesis. We have recently established an *fdx1b* zebrafish mutant line (*fdx1b*^*−/−*^) and showed that disruption of *fdx1b* leads to an impaired stress response and severe global cortisol deficiency in larvae [33].

Here, we explore the *in vivo* metabolic consequences of GC deficiency due to a disrupted mitochondrial ferredoxin system in the *fdx1b*^*−/−*^ mutant zebrafish line. The deficiency of multiple steps of GC biosynthesis and the resulting severity of GC deficiency in the *fdx1b*^*−/−*^ mutants make this model particularly well suited for such studies. A combination of transcriptomics and metabolic profiling in *fdx1b*^*−/−*^ mutants revealed an extensive reprogramming of metabolic pathways, including glutamine metabolism as well as significant changes in the linked glutathione and purine biosynthesis pathways. Remarkably, we identified post-transcriptional regulation of key enzymes underlying some of these changes. A comparison of the *fdx1b*^*−/−*^ mutants with a zebrafish model of secondary adrenal insufficiency (*rx3* strong) revealed both overlapping and distinct transcriptional and metabolic changes in these two models of GC deficiency. Finally, blood samples from individuals with primary adrenal insufficiency showed altered amino acid concentrations consistent with the metabolic alterations in our *fdx1b*^*−/−*^ mutants, suggesting a translational relevance to humans with GC deficiency.

## 2 Materials and methods

### 2.1 Zebrafish husbandry

Adult zebrafish (AB wild-type strain) were raised and bred according to standard methods [79]. Embryos were obtained by natural spawning and incubated at 28.5 °C in 1× E3 medium (5 mmol/l NaCl, 0.17 mmol/l KCl, 0.33 mmol/l CaCl_2_, 0.33 mmol/l MgSO_4_). The developmental stages were determined in hours post-fertilization (hpf) as previously described [41]. All procedures were approved by the Home Office, United Kingdom and carried out in line with the Animals (Scientific Procedures) Act 1986.

### 2.2 Phylogenetic analysis

The protein sequences of the examined genes (Table S1) were retrieved from ENSEMBL v84 (GRCz10) and phylogenetic analysis was carried out as previously reported [40].

### 2.3 Treatment and sampling

*fdx1b*^*−/−*^ mutants were identified due to their impaired visual background adaptation (VBA) at 96 hpf as previously described [33]. Larvae (96 hpf) were exposed for 24 h with 25 μM Dexamethasone (DEX; Sigma-Aldrich, #D1756) in E3 medium supplemented with 0.1% DMSO. Wild-type embryos/larvae were treated with 1 mM of the glutaminase inhibitor 6-Diazo-5-oxo-L-norleucine (DON; Sigma-Aldrich, #D2141) in E3 medium at 72 hpf and 96 hpf for 24 h and 48 h, respectively. For subsequent processing, larvae were either snap frozen in liquid nitrogen at 120 hpf for RNA extraction or fixed in 4% paraformaldehyde for whole-mount *in situ* hybridization.

### 2.4 Total RNA extraction

20 larvae were sampled and homogenized in QIAzol lysis reagent (Qiagen, # 79306) and stored overnight at −80 °C. Samples were then passed several times through a syringe (BD Microlance, 0.5 × 25 mm, #3086982), and RNA was extracted using the RNeasy Plus Universal Kit (Qiagen, #73442) according to the manufacturer's instructions. The integrity and quality of the total RNA was checked on an agarose gel and NanoDrop spectrometer (Thermo Scientific). Only RNA with A260/280 ratio ≥ 2 and A260/230 ratio ≥ 1 was used for subsequent analysis.

### 2.5 cDNA synthesis and quantitative RT-PCR (qRT-PCR)

cDNA synthesis was carried out with 1 μg RNA using the SuperScript VILO cDNA Synthesis Kit (LifeTechnologies, #11754-050). Expression levels of the examined genes were examined with Power SYBRGreen PCR Master Mix (Thermofisher, #4367659) according to the manufacturers protocol. Primer sequences for the examined genes are listed in Table S2.

### 2.6 Whole-mount in situ hybridization

Both the generation of probes using gene-specific oligos summarized in Table S3 and whole-mount *in situ* hybridizations were carried out as previously described [77]. *cyp17a2* expression was used to determine the size of the interrenal gland using Image J software.

### 2.7 Human study

Five participants with primary adrenal insufficiency followed at the pediatric endocrinology clinic at Kanuni Sultan Süleyman Education and Research Hospital were enrolled. All patients were on regular hydrocortisone treatment before the study. Patients were admitted to the hospital and monitored for general well-being, heart rate, blood pressure and blood sugar. Plasma samples were obtained after an overnight fast between 7 and 9 a.m. at on-treatment state. After 48 h of discontinuation of hydrocortisone treatment plasma sampling was repeated at fasting and off-treatment state at 7–9 a.m. Plasma samples were frozen and stored at −80 °C until further analysis. Cortisol-sufficient (“On-treatment”) and cortisol-deficient (“Off-treatment”) states in patients were monitored by measuring plasma ACTH concentrations. No adverse events related to discontinuation of treatment for 48 h in the hospital settings were observed. The study design was approved by the local ethical committee (Approval number 10840098–604.01.01−E.4622). Written consent was obtained from the families of the participating patients.

### 2.8 Next generation sequencing (RNA-seq)

RNA of 20 larvae (120 hpf; three biological replicates) was extracted as described above. RNA integrity was checked with a 2100 Bioanalyzer (Agilent). cDNA libraries were generated using the TruSeq Stranded Total RNA Sample Prep Kit with the Ribo-Zero Gold depletion set (Illumina) following the manufacturer's protocol. The libraries were sequenced on the Illumina Hiseq 2500 as single-end 50 base. Image analysis and base calling were performed using RTA 1.18.61 and bcl2fastq 1.8.2.

### 2.9 Metabolic profiling

#### 2.9.1 ^1^H NMR spectroscopy

The metabolic study using NMR-Spectroscopy was carried out with *fdx1b*^*−/−*^ and wild-type siblings larval extracts (25 larvae/sample; at least four biological replicates) as described in detail in [74].

#### 2.9.2 PLC-FCS and IC-CD

Adenosine compounds, thiols and free amino acids were extracted from 30 larvae (120 hpf, in five biological replicates) with 0.3 ml of 0.1 M HCl in an ultrasonic ice-bath for 10 min. The resulting homogenates were centrifuged twice for 10 min at 4 °C and 16.400 g to remove cell debris. Adenosines were derivatized with chloroacetaldehyde as previously described [9] and separated by reversed phase chromatography on an Acquity BEH C18 column (150 mm × 2.1 mm, 1.7 μm, Waters) connected to an Acquity H-class UPLC system. Prior separation, the column was heated to 42 °C and equilibrated with 5 column volumes of buffer A (5.7 mM TBAS, 30.5 mM KH2PO4 pH 5.8) at a flow rate of 0.45 ml min-1. Separation of adenosine derivates was achieved by increasing the concentration of buffer B (2/3 acetonitrile in 1/3 buffer A) in buffer A as follows: 1 min 1% B, 1.6 min 2% B, 3 min 4.5% B, 3.7 min 11% B, 10 min 50% B, and return to 1% B in 2 min. The separated derivates were detected by fluorescence (Acquity FLR detector, Waters, excitation: 280 nm, emission: 410 nm, gain: 100) and quantified using ultrapure standards (Sigma). Determination of amino acid levels was done as described in Weger et al. [74]. Total glutathione was quantified by reducing disulfides with DTT followed by thiol derivatization with the fluorescent dye monobromobimane (Thiolyte, Calbiochem). For quantification of GSSG, free thiols were first blocked by NEM followed by DTT reduction and monobromobimane derivatization. GSH equivalents were calculated by subtracting GSSG from total glutathione levels. Derivatization was performed as described in Wirtz et al. [82]. UPLC-FLR analysis was carried out using the system described above. Separation was carried out using the above described UPLC-FLR system with a binary gradient of buffer A (100 mM potassium acetate, pH 5.3) and solvent B (acetonitrile) with the following gradient: 0 min 2.3% buffer B; 0.99 min 2.3%, 1 min 70%, 1.45 min 70%, and re-equilibration to 2.3% B in 1.05 min at a flow rate of 0.85 ml min-1. The column (Acquity BEH Shield RP18 column, 50 mm × 2.1 mm, 1.7 μm, Waters) was maintained at 45 °C and sample temperature was kept constant at 14 °C. Monobromobimane conjugates were detected by fluorescence at 480 nm after excitation at 380 nm after separation.

For absolute quantification by HPLC of amino acids in human blood samples, fluorescence derivatisation followed by separation with an Acquity H-class UPLC system (Waters) and fluorescence detection was employed. Data acquisition and processing was performed with the Empower3 software suite (Waters). Organic acids were quantified by ion chromatography and conductivity detection after cation suppression with an ICS-3000 system (Dionex). Data acquisition and processing was performed with the Chromeleon 6.7 software (Dionex).

### 2.10 Data analysis

#### 2.10.1 RNA-seq data processing and analysis

Single-end reads were mapped onto the zebrafish genome (GRCz10) using STAR 2.3.8 [17]. A custom Perl script was used to count uniquely mapped reads for each annotated gene locus (ENSEMBL v84) at both exonic and intronic regions as described [4]. Data was analysed using DESeq2 [54]. RNA-seq raw data for the *rx3* mutants was retrieved from our previous study [74] available at NCBI's Gene Expression Omnibus [20] (GSE76073) and reanalysed to assess differential gene expression between *rx3* strong (lack of eyes and GC-deficient) and *rx3* weak mutants (only lack of eyes). To assess the interaction between differential expressed genes of *fdx1b*^*−/−*^ mutants and *rx3* mutants, we applied a model to the counts of each gene: ~phenotype + mutated_gene + time + phenotype:mutated_gene (where mutated_gene is *rx3* or *fdx1b*, phenotype is wild-type or mutant and time is the Zeitgeber time). To determine the statistical significance for the interaction term, we used a likelihood ratio test to compare the full model and a reduced model that contains all experimental factor of the full model excluding the interaction term (phenotype:mutated_gene).

Total RNA sequencing allows the quantification not only of reads mapped to exons (mRNA) but also of those mapped to introns (pre-mRNA) [4,26]. To assess changes in mRNA and pre-mRNA level between *fdx1b*^*−/−*^ mutants and wild-type siblings, we applied the exon-intron split analysis (EISA) described in [25]. The exon/intron ratio was used as a proxy for relative mRNA half-life. Additional publicly available RNA-seq based expression data [12,18,81] were analysed using DESeq2 [54].

#### 2.10.2 Gene set enrichment analysis

Gene sets were retrieved from GO ontology [64], KEGG based metabolic pathways manually redefined for zebrafish [74], Ingenuity Pathway Analysis (Qiagen) and MSigDB C2 canonical pathways [49]. We defined the gene set of ACTH targets from [84]. To perform gene set enrichment analysis for differentially expressed genes and interaction between *rx3* strong and *fdx1b*^*−/−*^ mutants, we employed the *camera* function of the limma package [83] using linear model experimental factors detailed in RNA-seq data processing and analysis. Gene set enrichment analysis was visualized using barcode plots of the *limma* package [83]. Briefly, t-stats from the linear model were ranked from largest to smallest (from left to right). The position of the chosen gene set are marked by vertical bars representing a barcode. The relative enrichment of the vertical bars is depicted by enrichment worm above the barcode.

#### 2.10.3 NMR based data and metabolic profiling

Data were log transformed for HPLC based methods or a variance stabilizing transformation was applied to the data from the NMR based measurements [42]. We subsequently fit a linear model ~genotype*treatment for each metabolite (HPLC based) or each peak (NMR) (Table S4). Metabolite differences between patients under On- and Off-treatment conditions were assessed using a linear mixed-effects model. To this end, a full model was fitted to the data: y ~ age + treatment + (1|ID), where y is the log2 normalized serum concentration with a specific age at treatment condition (On/Off). 1|ID represents a patient specific random effect on the baseline. The full model was compared to a reduced model (y ~ age + (1| ID)) using a likelihood ratio test.

#### 2.10.4 Multiple testing

All *p*-values presented in this manuscript were corrected for multiple testing using the method of Benjamini-Hochberg [7], if applicable.

### 2.11 Data availability

The data generated for this publication have been deposited in NCBI's Gene Expression Omnibus [20] and are accessible through GEO Series accession number GSE107547 and are available in Table S5. Reanalysed *rx3* strong data are available in Table S6.

## 3 Results

### 3.1 Disruption of ferredoxin leads to profound transcriptional alterations in several metabolic pathways

**Fig. 1:**
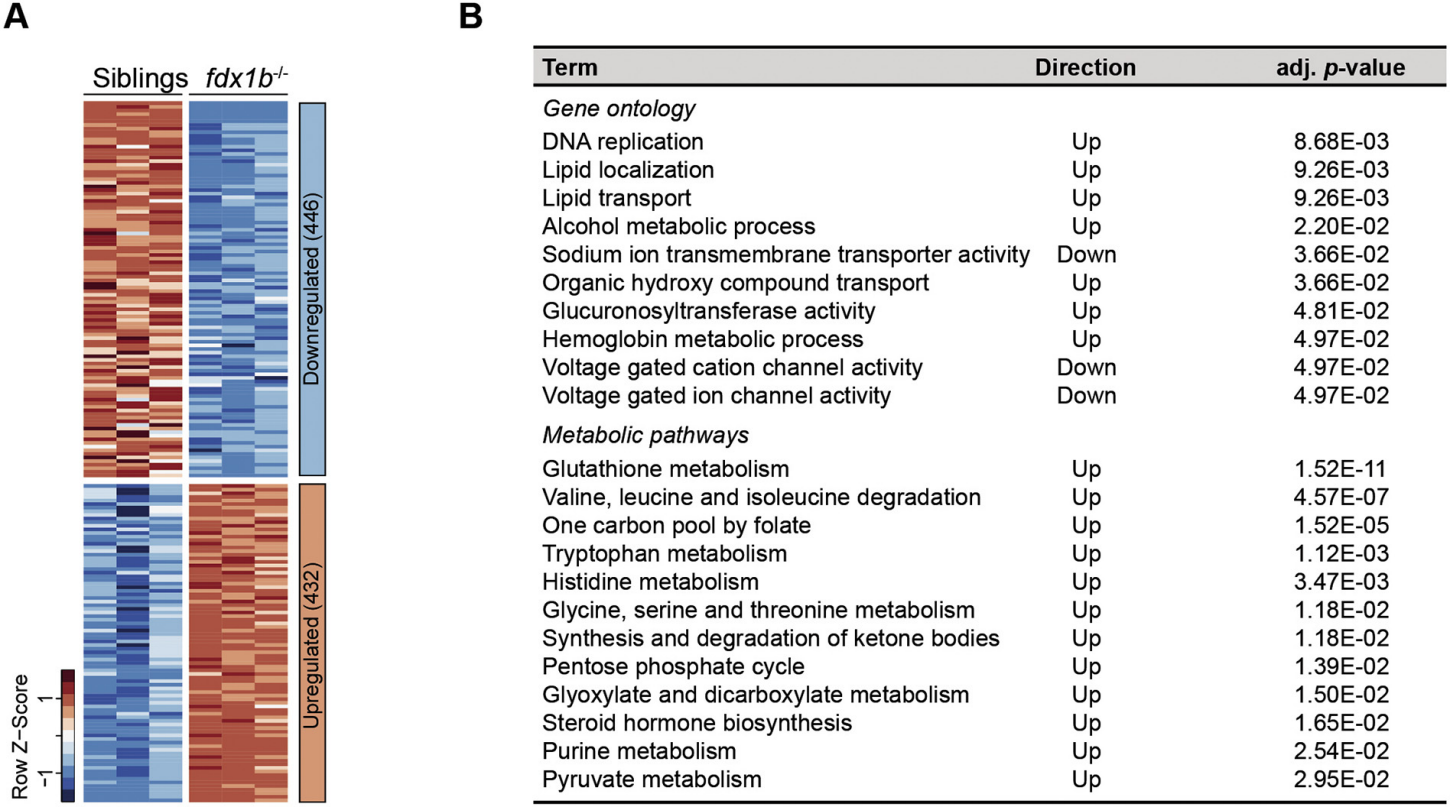
*fdx1b*^*−/−*^ mutants show an extensive transcriptional reprogramming of energy and biomolecule generating metabolic pathways. (A) Heatmap of normalized mRNA expression levels of genes in *fdx1b*^*−/−*^ mutant larvae and wild-type siblings. Red, high expression; blue, low expression. From in total 878 differentially expressed genes, 446 genes were down-regulated, and 432 genes were up-regulated in *fdx1b*^*−/−*^ mutant larvae. (B) Gene set enrichment analysis of differentially expressed genes (*fdx1b*^*−/−*^*vs.* wild-type sibling larvae). Direction indicates whether the up- or down-regulated genes in *fdx1b*^*−/−*^ mutants are enriched for the indicated term.

The potential dysregulation of gene expression as a consequence of GC deficiency in *fdx1b*^*−/−*^ mutants was analysed by RNA-seq from total RNA obtained from *fdx1b*^*−/−*^ and wild-type sibling larvae at 120 hpf. The RNA-seq showed a high biological reproducibility as demonstrated by the strong correlation between biological replicates (*R* ≥ 0.0993; Fig. S1A). Overall, the differential gene expression analysis identified a down-regulation of 446 and an up-regulation of 432 genes in the *fdx1b*^*−/−*^ larvae (adjusted *p*-value ≤.01, |log2Fold change| ≥ 0.25; Fig. 1A and Table S5). By employing an enrichment analysis based on gene ontology categories the most prominent functions in the *fdx1b*^*−/−*^ gene set were identified. Down-regulated genes in *fdx1b*^*−/−*^ larvae were enriched in pathways for ion transport including sodium ion transmembrane transporter activity, voltage gated cation and ion channel activity (Fig. 1B). These findings appear to be consistent with the role of GCs in the osmoregulation in fish larvae [71]. In contrast, we observed an enrichment of up-regulated genes in a broad range of metabolic pathways in *fdx1b*^*−/−*^ mutants. This included pathways of lipid localization and transport, organic hydroxy compound transport as well as processes of alcohol, co-factor and haemoglobin metabolism (Fig. 1B). In addition, up-regulated genes were enriched in DNA replication and cell cycle associated pathways (Fig. 1B and Table S7), supporting the observed importance of GCs for circadian regulation of cell proliferation in zebrafish larvae [16]. A further more specific analysis of metabolic pathways based on KEGG annotation [74] identified an expected compensatory up-regulation of genes involved in steroid hormone synthesis in response to the disrupted steroidogenesis (Fig. 1B). In addition, an enrichment of pathways of energy metabolism was detected, which included the pentose phosphate cycle, pyruvate metabolism and synthesis as well as ketone degradation (Fig. 1B). Also, pathways leading to the degradation of the amino acids valine, leucine, and isoleucine and the metabolism of tryptophan, histidine, glycine, serine, and threonine were found to be up-regulated. In addition, we found an up-regulation of the folate mediated one‑carbon metabolic pathway, of glyoxylate and dicarboxylate metabolism, of purine metabolism, and of glutathione metabolism (Fig. 1B). Overall, the systemic GC deficiency in *fdx1b*^*−/−*^ mutant larvae resulted in an extensive transcriptional reprogramming of genes involved in a widespread set of metabolic pathways. In addition to anticipated pathways directly or indirectly linked to energy metabolism, also crucial biosynthetic pathways such as purine biosynthesis and the redox-buffering glutathione metabolism were affected.

### 3.2 *fdx1b*^*−/−*^ larvae exhibit specific glucocorticoid-dependent metabolic alterations

**Fig. 2:**
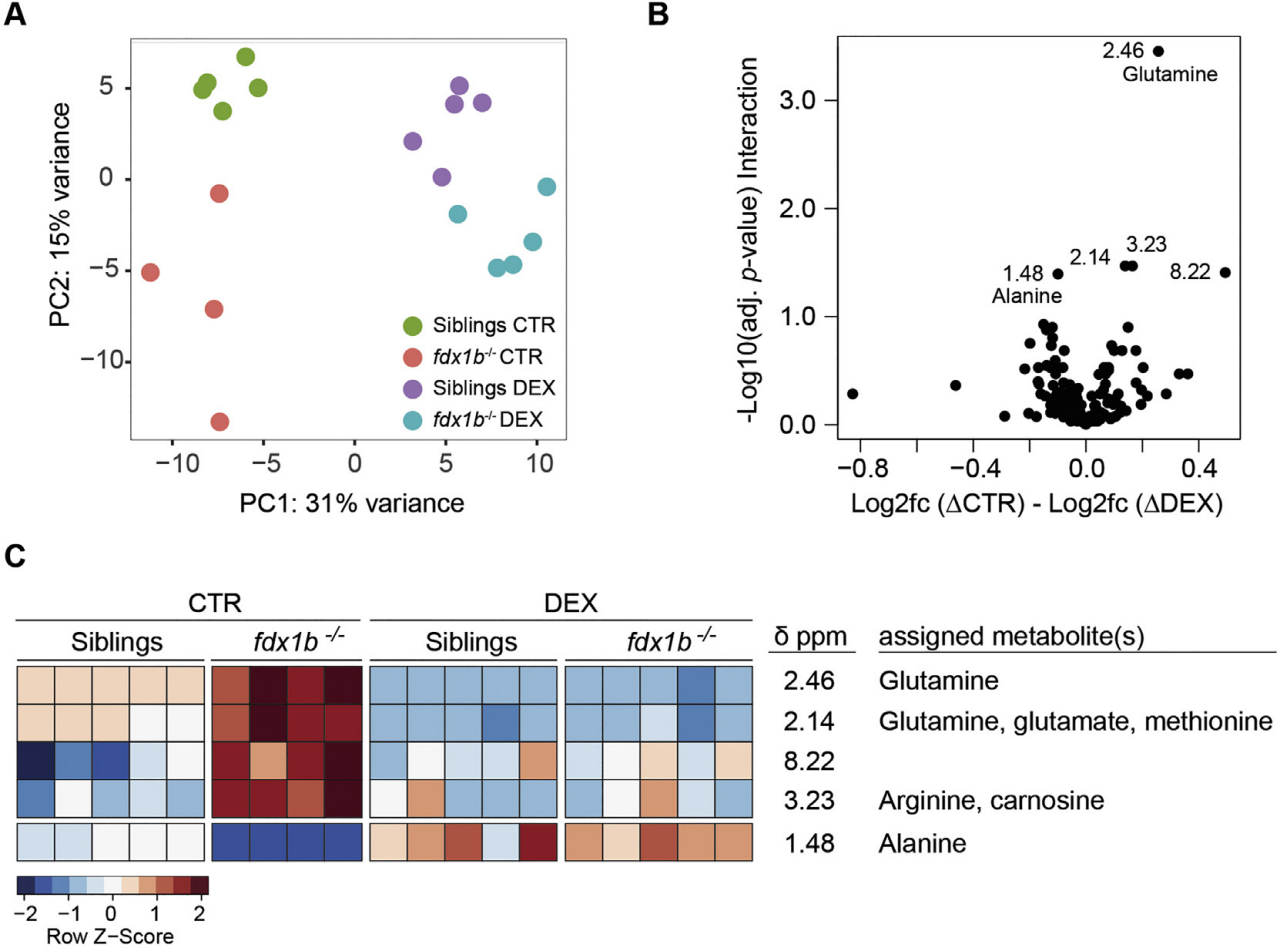
Untargeted ^1^H NMR spectroscopy analysis reveals changes in the metabolome of *fdx1*^*−/−*^ mutant larvae. (A) Principal component analysis (PCA) score plots of the ^1^H NMR spectra of *fdx1b*^*−/−*^ mutant and wild-type sibling larvae treated with dexamethasone (DEX) or vehicle as control (CTR). (B) Volcano plot represents the difference in fold change for each peak between *fdx1b*^*−/−*^ and wild-type sibling larvae under DEX and CTR treatment. Significant peaks are labeled. (C) Heatmap of peaks with a significant interaction. Red = high expression; blue = low expression. The assigned metabolites including glutamine and alanine are altered in *fdx1b*^*−/−*^ mutant larvae under basal conditions, but not upon DEX treatment.

**Fig. 3:**
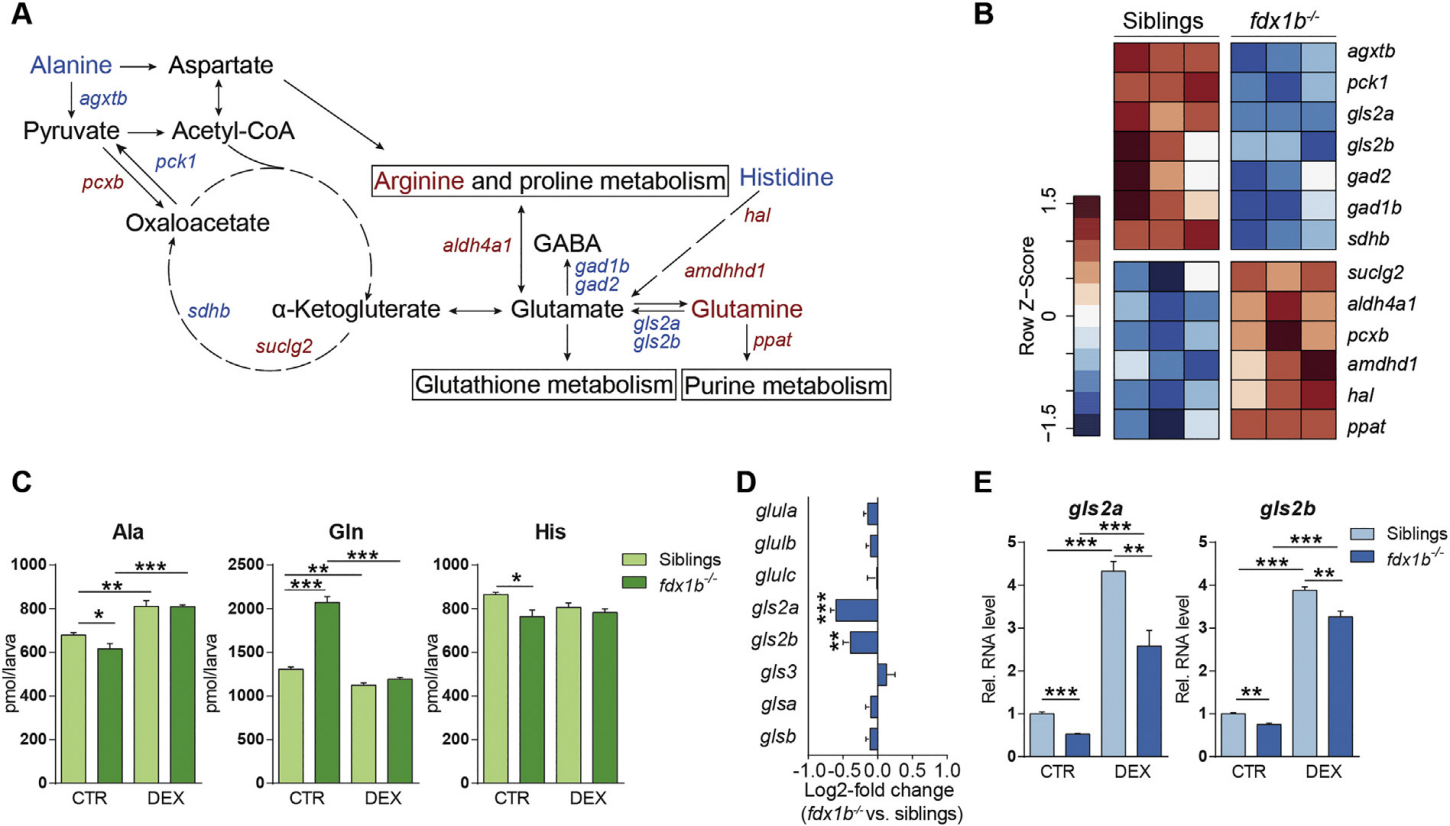
*fdx1b*^*−/−*^ mutant larvae exhibit alterations in gene expression related to alanine, aspartate and glutamate metabolism and in glutamine-family amino acids. (A) Schematic represents “alanine, aspartate and glutamate metabolism”, and glutamine-family amino acids. Altered metabolites and genes in *fdx1b*^*−/−*^ larvae are indicated in red for up-regulation and blue for down-regulation. (B) Heatmap showing differentially expressed genes of alanine, aspartate and glutamate metabolism in *fdx1b*^*−/−*^ mutant larvae. (C) HPLC-based measurements of metabolite levels of alanine (Ala), glutamine (Gln) and histidine (His) in *fdx1b*^*−/−*^ mutant larvae and wild-type siblings in the in the absence (CTR) or presence of dexamethasone (DEX). (D) Fold change of glutamate metabolism genes of *fdx1b*^*−/−*^*vs.* wild-type sibling larvae. (E) qRT-PCR analysis of the zebrafish *gls2a* and *gls2b* in *fdx1b*^*−/−*^ mutant larvae and wild-type siblings (120 hpf) in the absence (CTR) or presence of dexamethasone (DEX).

An untargeted ^1^H NMR spectroscopy analysis with aqueous larval extracts of *fdx1b*^*−/−*^ mutants and wild-type siblings was performed to assess if observed transcriptional changes in *fdx1b*^*−/−*^ mutants affect metabolite concentrations. Principal component analysis (PCA) indicated clear differences in the metabolome between the *fdx1b*^*−/−*^ mutants and their wild-type siblings (Fig. 2A). To understand the GC-dependent metabolic changes in *fdx1b*^*−/−*^ mutants, we also analysed larvae treated with the synthetic GC dexamethasone (DEX). DEX treatment led to a general shift of the ^1^H NMR spectra along principal component (PC) 1, and a closer clustering of the *fdx1b*^*−/−*^ mutant with the control metabolome along PC2 (Fig. 2A). This suggests that GC treatment can rescue some of the changes observed in *fdx1b*^*−/−*^ larvae. To identify the main metabolites altered by GC deficiency in *fdx1b*^*−/−*^ larvae, we calculated the ratio of changes in mutants in the absence and presence of DEX for each NMR feature. This ratio indicates the extent of altered differences by GC treatment between *fdx1b*^−/−^ larvae and wild-type siblings. The statistical significance of this ratio was determined by calculating the interaction term between treatment and genotype. Following this approach, we identified several significantly altered peaks, including those at 2.46 ppm and 1.48 ppm corresponding to glutamine and alanine, respectively (Fig. 2B and C). The abundance of glutamine increased, whereas alanine decreased in untreated *fdx1b*^*−/−*^ larvae. The intensities of all identified peaks were different to the wild-type in untreated *fdx1b*^*−/−*^ mutants, whereas wild-type and *fdx1b*^*−/−*^ mutants showed similar patterns after DEX treatment (Fig. 2C), indicating GC-dependent rescue of the differences. Notably, both glutamine and alanine can serve as substrates for gluconeogenesis [10], and thus are highly relevant for endogenous glucose production. Glutamine also acts as key nitrogen source for the synthesis of biomolecules such as nucleotides and other amino acids [13]. Therefore, the observed alterations in these amino acids indicate a dysregulation of energy metabolism and synthesis of biomolecules in GC-deficient *fdx1b*^*−/−*^ larvae and confirm the relevance of observed changes detected on transcriptomic level. By using targeted HPLC-based metabolic profiling, we expanded the assessment of metabolites to other amino acids and nucleotides (Table S4). This analysis confirmed the GC-dependent decrease in alanine and the increase in glutamine concentrations (Fig. 3A and C). In addition, histidine, which is degraded in a pathway eventually leading to the formation of glutamate, shows decreased concentrations in *fdx1b*^*−/−*^ mutants (Fig. 3C). Expression of the genes encoding for the enzymes involved in these linked pathways broadly correlated with observed metabolite changes in *fdx1b*^*−/−*^ mutant larvae (Fig. 3A and B).

### 3.3 Glutamine metabolism and linked pathways are affected in *fdx1b*^*−/−*^ mutant larvae

Our explorative and untargeted analyses implied a key role of the metabolism of glutamine as a dysregulated pathway by GC deficiency. As glutamine and glutamate is linked with other major affected pathways including energy metabolism as well as glutathione and purine metabolism, we focussed on the metabolism of glutamine and glutamate. In mammals, glutamine synthetase (GLUL) is responsible for the synthesis of glutamine from glutamate, whereas two glutaminases (GLS and GLS2) are responsible for glutaminolysis of glutamine into glutamate [2] (Fig. S2A). *In silico*, three paralogs of glutamine synthetase (Glula, Glulb, Glulc), two paralogs of glutaminase (Glsa, Glsb) and two paralogs of glutaminase 2 (Gls2a, Gls2b) as well as an additional novel Ensembl prediction termed Glsl were identified in zebrafish. The phylogenetic analysis demonstrates clustering of the zebrafish glutamine synthetase and glutaminase proteins, except for Glsl, with their respective orthologs in humans, mice and rats (Fig. S2B). This finding suggests a conserved function of glutamine metabolism between vertebrates. Only expression of the glutaminase *gls2a* and *gls2b* genes, but not of the *glul* genes, was significantly different between *fdx1b*^*−/−*^ larvae and wild-type siblings (Fig. 3D and Table S5). Since zebrafish larvae are not free feeding at the examined stage, increased glutamine concentrations in *fdx1b*^*−/−*^ mutants are likely to be caused by impaired glutaminolysis due to reduced expression and function of glutaminases (*gls2a* and *gls2b*). qRT-PCR of wild-type and *fdx1b*^*−/−*^ larvae raised in the absence or presence of DEX demonstrated a significant down-regulation of both *gls2a* and *gls2b* in *fdx1b*^*−/−*^ larvae with an up-regulation in response to DEX treatment (Fig. 3E). This finding suggests GC-dependent gene expression of the *gls2a* and *gls2b* genes. To better understand the physiological relevance of the affected glutaminase paralogs, the spatio-temporal expression of *gls2a*, *gls2b*, *glsl*, *glsa* and *glsb* was analysed. The reanalysis of a previously published developmental transcriptome data set including data from zygote stage to 120 hpf [81] showed that all glutaminases were expressed in larvae at 120 hpf with a maternal contribution mainly for *glsb* and *gls2a* (Fig. S2C). Whole-mount *in situ* hybridization in 120 hpf wild-type larvae showed specific *gls2a* expression in the liver, whereas *gls2b* is expressed in both liver and intestine (Fig. S2D). The other paralogs were mainly expressed in the larval brain (*glsa*, *glsb*) or in the swim bladder (*glsl*, *glsb*) (Fig. S2D). These data suggest that increased glutamine concentrations in *fdx1b*^*−/−*^ mutant larvae is resulting from impaired glutaminolysis due to impaired GC-dependent transcription of liver and intestine specific glutaminases (*gls2a* and *gls2b*).

### 3.4 *fdx1b*^*−/−*^ mutants show profound changes in glutathione metabolism and markers of oxidative stress

**Fig. 4:**
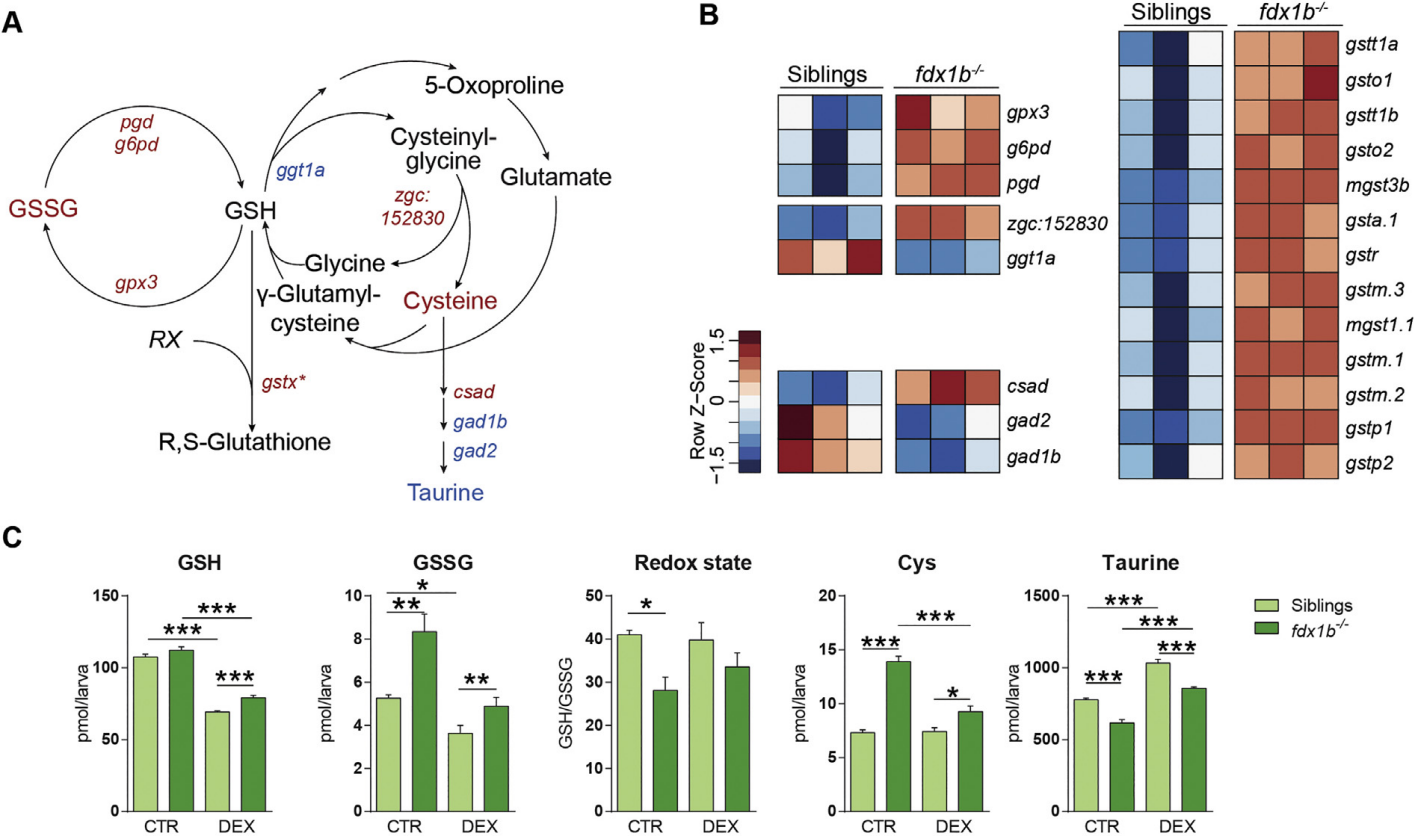
Dysregulations in glutathione metabolism and markers of oxidative stress in *fdx1b*^*−/−*^ mutant larvae are only partially caused by glucocorticoid-deficiency. (A) Schematic illustrates “glutathione metabolism”. Metabolites and genes of this pathway altered in *fdx1b*^*−/−*^ larvae are marked in red for up-regulation and blue for down-regulation. (B) Heatmap showing differentially expressed genes of glutathione metabolism in *fdx1b*^*−/−*^ mutant larvae. (C) Metabolite levels of cysteine (Cys), reduced (GSH) and oxidized (GSSG) glutathione, taurine and the GSH/GSSG ratio as a measure of oxidative stress in *fdx1b*^*−/−*^ mutant and wild-type sibling larvae in the absence (CTR) or presence of dexamethasone (DEX).

The most enriched metabolic pathway in the *fdx1b*^*−/−*^ mutants was glutathione metabolism (Fig. 1B; adj. *p*-value = 1.52E-11). Glutathione (GSH) is an antioxidant and a cellular signalling molecule, which is used by glutathione S-transferases (GSTs) to detoxify reactive oxygen species (ROS) [22]. The vast majority of GSTs were up-regulated in *fdx1b*^*−/−*^ mutants (Fig. 4A and B) suggesting increased oxidative stress levels in *fdx1b*^*−/−*^ larvae. On the biochemical level, increased concentrations of glutathione disulphide (GSSG) and a decrease in the GSH to GSSG ratio as marker of increased oxidative stress [22] were observed in the *fdx1b*^*−/−*^ mutants (Fig. 4C). These findings are further supported by a decrease of the antioxidant taurine and an increase of cysteine in *fdx1b*^*−/−*^ larvae (Fig. 4C). In addition, the majority of Nrf2 (nuclear factor E2-related factor 2) target genes were up-regulated in *fdx1b*^*−/−*^ larvae (Fig. S3A and B). Since Nrf2 is the master regulator of genes mediating the response to oxidative stress [22], this finding is highly suggestive for systemically elevated oxidative stress levels in *fdx1b*^*−/−*^ mutants. Importantly, DNA damage caused by oxidative stress has been implicated in human pathology [69]. Consistent with the detected increased levels of oxidative stress in *fdx1b*^*−/−*^ mutants, genes implicated in double-strand break induced DNA repair (*i.e.*, nonhomologous end joining and homologous recombination) are significantly up-regulated in *fdx1b*^*−/−*^ mutants (Fig. S3C and D).

Remarkably, the increased transcript levels of the Nrf2 key target gene *fth1a* [11] and the antioxidant gene *duox* returned to wild-type levels by DEX treatment in *fdx1b*^*−/−*^ larvae (Fig. S3E), indicating an important role of GCs in the altered oxidative stress levels. However, on the biochemical level, GSH, GSSG and taurine concentrations remained significantly different between wild-type and mutant larvae after DEX treatment (Fig. 4C), indicating that GC deficiency might not be the only cause of altered oxidative stress levels in *fdx1b*^*−/−*^ mutants.

### 3.5 Expression of key enzymes of *de novo* purine synthesis pathway is regulated by glucocorticoids

**Fig. 5:**
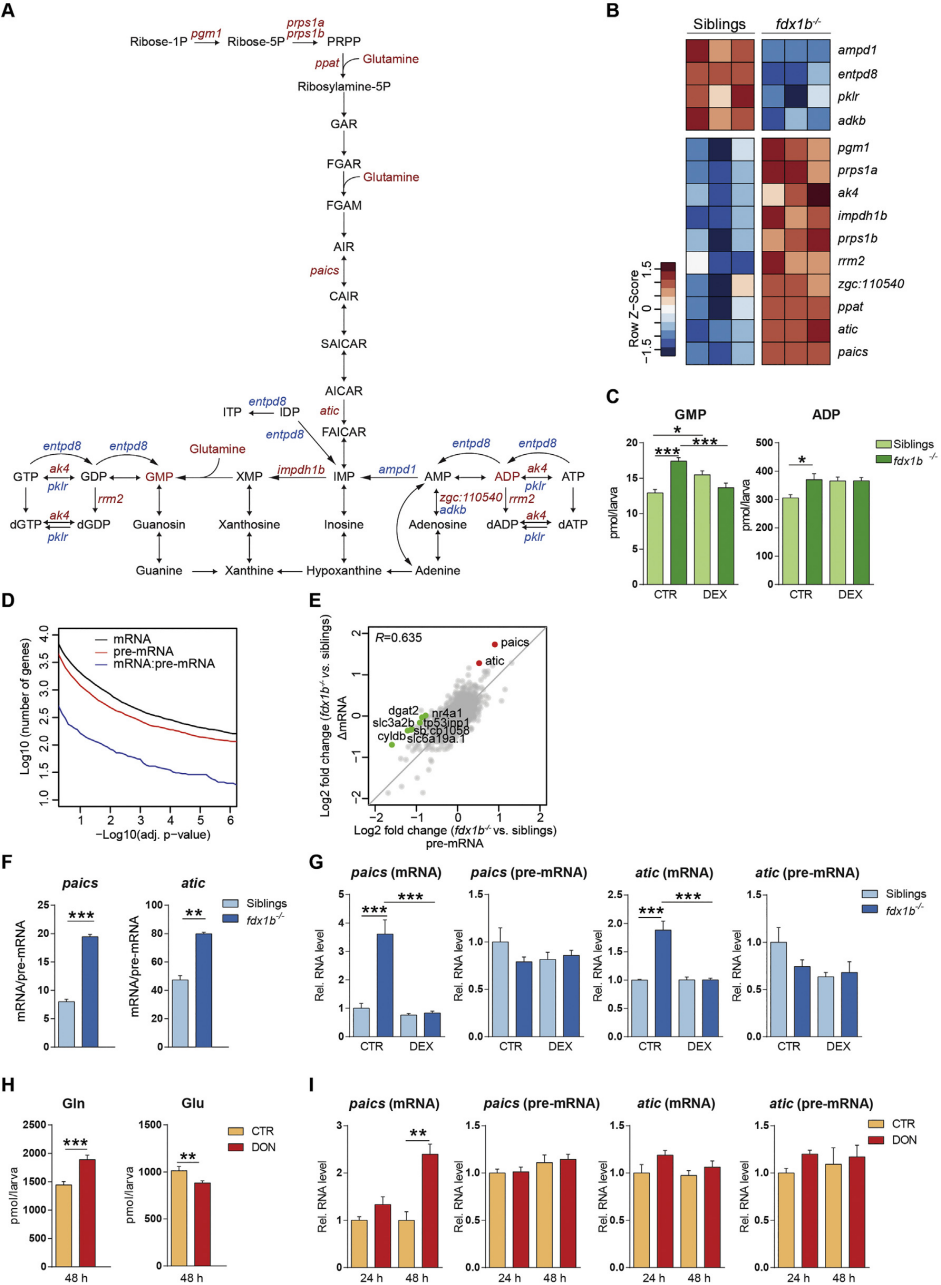
Glucocorticoids regulate *de novo* purine synthesis at a post-transcriptional level. (A) Schematic of the “purine metabolism” pathway. Metabolites and genes of this pathway altered in *fdx1b*^*−/−*^ mutant larvae are marked in red for up-regulation and blue for down-regulation. (B) Heatmap showing differentially expressed genes of purine metabolism in *fdx1b*^*−/−*^ mutant larvae. (C) Metabolite levels of guanosine monophosphate (GMP) and adenosine diphosphate (ADP) in *fdx1b*^*−/−*^ mutant and wild-type sibling larvae (120 hpf) in the absence (CTR) or presence of dexamethasone (DEX). (D) Number of differentially expressed genes in function of the adjusted *p*-value grouped by the different levels at which they were assessed. mRNA (black), pre-mRNA (red), and genes that are differentially affected between mRNA and pre-mRNA (blue). (E) Comparison of pre-mRNA and mRNA expression changes in *fdx1b*^*−/−*^ mutant larvae. *R* indicates Pearson correlation. Significantly deregulated genes at the level of ∆exon-∆intron are labeled green. Predominantly post-transcriptional (∆exon>∆intron) altered genes are shown in red. (F) mRNA half-life approximation of *paics* and *atic* in *fdx1b*^*−/−*^ mutant and wild-type sibling larvae. (G) qRT-PCR analysis assessing mRNA and pre-mRNA levels of *paics* and *atic* in *fdx1b*^*−/−*^ mutant and wild-type sibling larvae (120 hpf) in the absence (CTR) or presence of dexamethasone (DEX). (H, I) Glutamine (Gln) and glutamate (Glu) levels (H) and transcript levels of *paics* and *atic* (I) in wild-type larvae treated with the glutaminase inhibitor 6-Diazo-5-oxo-L-nor-Leucine (DON) or vehicle as a control (CTR).

Another enriched metabolic pathway in the *fdx1b*^*−/−*^ mutant larvae was the glutamine-dependent *de novo* purine synthesis pathway (Fig. 1B; adj. *p*-value = 2.54E-2). In fact, two enzymes of this pathway, *paics* and *atic* (Fig. 5A and B), were among the five most highly up-regulated genes in the transcriptome analysis (Table S5). Interestingly, the only four additional differentially expressed genes of *de novo* purine synthesis leading to inosine monophosphate (IMP) were all significantly up-regulated in *fdx1b*^*−/−*^ mutants (Fig. 5A and B). Several enzymes of purine metabolism downstream of IMP also showed an altered mRNA abundance in *fdx1b*^−/−^ mutants (Fig. 5A and B), but no clear trend for global up- or down-regulation of these genes was detected. However, on the biochemical level, both guanosine monophosphate (GMP) and adenosine diphosphate (ADP) concentrations were significantly increased in *fdx1b*^*−/−*^ larvae (Fig. 5C). Differences between mutants and wild-type sibling larvae were decreased under DEX treatment, thus indicating the GC-dependency of these metabolites.

### 3.6 *paics* and *atic* are regulated by glucocorticoids in a post-transcriptional manner

The consequences of GC deficiency for altered post-transcriptional gene regulation were explored by employing an exon-intron split analysis (EISA) [4,26]. Due to the generally lower read coverage of intronic reads, the number of differentially expressed genes at pre-mRNA level is slightly lower than genes at mRNA level (Fig. 5D). The correlation between mRNA and pre-mRNA changes was high (Fig. 5E) indicating that differentially expressed genes in GC-deficient *fdx1b*^*−/−*^ larvae are predominantly regulated at the transcriptional level. To identify post-transcriptionally regulated genes, we aimed to identify genes showing significantly higher changes in mRNA level (ΔmRNA) than changes in transcription (Δpre-mRNA). By following this approach, *paics* and *atic* were identified to have positive and significant ΔmRNA/Δpre-mRNA ratios in *fdx1b*^*−/−*^ larvae (Fig. 5E). When using the mRNA/pre-mRNA ratio as a proxy for mRNA half-life [4], both *paics* and *atic* showed a significantly higher mRNA stability in *fdx1b*^*−/−*^ larvae than in wild-type siblings (Fig. 5F). Treatment of *fdx1b*^*−/−*^ larvae with DEX reduced the mRNA of *paics* and *atic* back to levels observed in wild-type without changing pre-mRNA levels (Fig. 5G), indicating the increased mRNA stability of *paics* and *atic* in *fdx1b*^*−/−*^ mutants is a consequence of GC deficiency. Given the importance of glutamine for *de novo* purine synthesis (Fig. 5A), we explored if the observed changes in glutamine metabolism with down-regulation of *gls2a* and *gls2b* in *fdx1b*^*−/−*^ mutants are linked to the altered mRNA stability of *paics* and *atic*. Therefore, Gls was pharmacologically inhibited using 6-Diazo-5-oxo-L-norleucine (DON) in wild-type larvae to mimic the alteration in glutamine metabolism observed in *fdx1b*^*−/−*^ mutants. 48 h of DON treatment significantly increased glutamine and decreased glutamate levels in wild-type larvae (Fig. 5H). Importantly, this treatment led to a selective increase in *paics* mRNA levels, whereas *atic* mRNA levels and the transcriptional rate of either gene remained unchanged (Fig. 5I).

To assess the relevance of glutamine concentrations in the observed effects, we analysed mRNA levels of *paics* and *atic* in a transgenic zebrafish model expressing an activated form of the Hippo pathway effector *yap1* [12]. Yap reprograms glutamine metabolism to increase nucleotide biosynthesis and these animals show increased GluI activity and subsequently elevated glutamine concentrations. Similar to the DON-treated larvae, only *paics* was differentially expressed in *yap1* transgenic zebrafish (Fig. S4A). Surprisingly, despite increased glutamine concentrations the levels of *paics* mRNA were significantly lower in *yap1* transgenic zebrafish than in wild-type (Fig. S4A). This observation suggests that glutamine accumulation is not directly resulting in increased *paics* mRNA stability.

miRNAs are a prominent class of post-transcriptional regulators [34]. Therefore, we analysed differential gene expression of miRNAs between *fdx1b*^*−/−*^ mutant and wild-type sibling larvae which we were able to detect in the total RNA-seq data set. *dre-mir-2192* was the only detected miRNA, which was up-regulated in *fdx1b*^*−/−*^ mutant larvae (adjusted *p*-value = .02; Fig. S4B). To test whether miRNAs affect gene expression of *atic* and *paics*, we re-analysed a previously published RNA-seq data set of a murine DICER knock-out model with a liver-specific inactivation of miRNA biogenesis [18]. A small decrease in transcription and mRNA abundance of *paics* in DICER knock-out mice was observed (Fig. S4C), whilst *atic* mRNA abundance showed a stronger down-regulation. *atic* transcription as indicated by pre-mRNA abundance was not as strongly reduced as mRNA abundance (interaction *p*-value = .03; Fig. S4C), suggesting that *atic* is post-transcriptionally regulated by miRNAs. These data are consistent with a stabilization of *atic* mRNA due to miRNA dependent effects also in the zebrafish. Altogether, *fdx1b*^*−/−*^ larvae showed a pervasive reprogramming of purine metabolism, leading to changes of purine metabolite concentrations. In addition, we provide the first evidence for GC-dependent post-transcriptional regulation of key factors in purine synthesis.

**Fig. 6:**
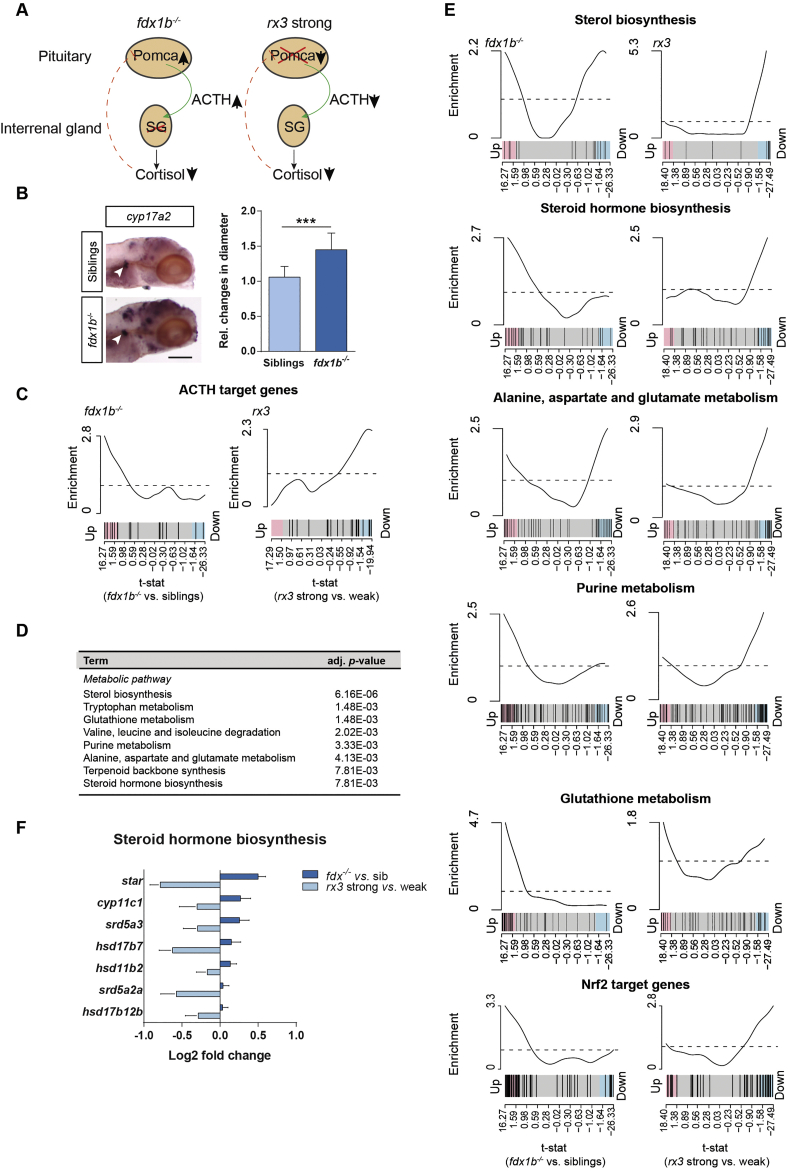
*fdx1b*^*−/−*^ mutant larvae exhibit hallmarks of primary adrenal insufficiency and differ from *rx3* strong mutant larvae reminiscent to secondary adrenal insufficiency. (A) Schematic shows the different alterations in the pituitary and interrenal gland axis of *fdx1b*^*−/−*^ and *rx3* strong mutant larvae. Abbreviations: Pomca, pro-opiomelanocortin; ACTH, adrenocorticotropin; SG, steroidogenesis. (B) Whole-mount *in situ* hybridization of *cyp17a2* to visualize the interrenal gland (arrow) in *fdx1b*^*−/−*^ mutant larvae (*n* = 17) and wild-type siblings (*n* = 11) at 120 hpf stage. Bar plot shows the relative (rel.) changes in the diameter of the interrenal gland size. (C) Barcode plots for ACTH target genes in *fdx1*^*−/−*^ mutant larvae (left) and *rx3* strong mutant larvae (right). The two mutant types show opposing directions in the gene set enrichment (*p*-value = 1.7 E−04) for gene set enrichment analysis. (D) Gene set enrichment analysis of differentially expressed genes showing different regulation between *fdx1b*^*−/−*^ and *rx3* strong. (E) Barcode plots for metabolic pathways and Nrf2 target genes in *fdx1b*^*−/−*^ mutant larvae (left) and *rx3* strong mutant larvae (right) in comparison to the corresponding control (wild-type siblings or *rx3* weak). (F) Fold changes of differentially expressed genes in steroid hormone biosynthesis between mutant larvae and their corresponding controls. The data for the *rx3* mutants is a reanalysis from our previously published data set [74]. Scale bar = 110 μm. *, *p* < .05, **, *p* < .01, ***, *p* < .001.

**Fig. 7:**
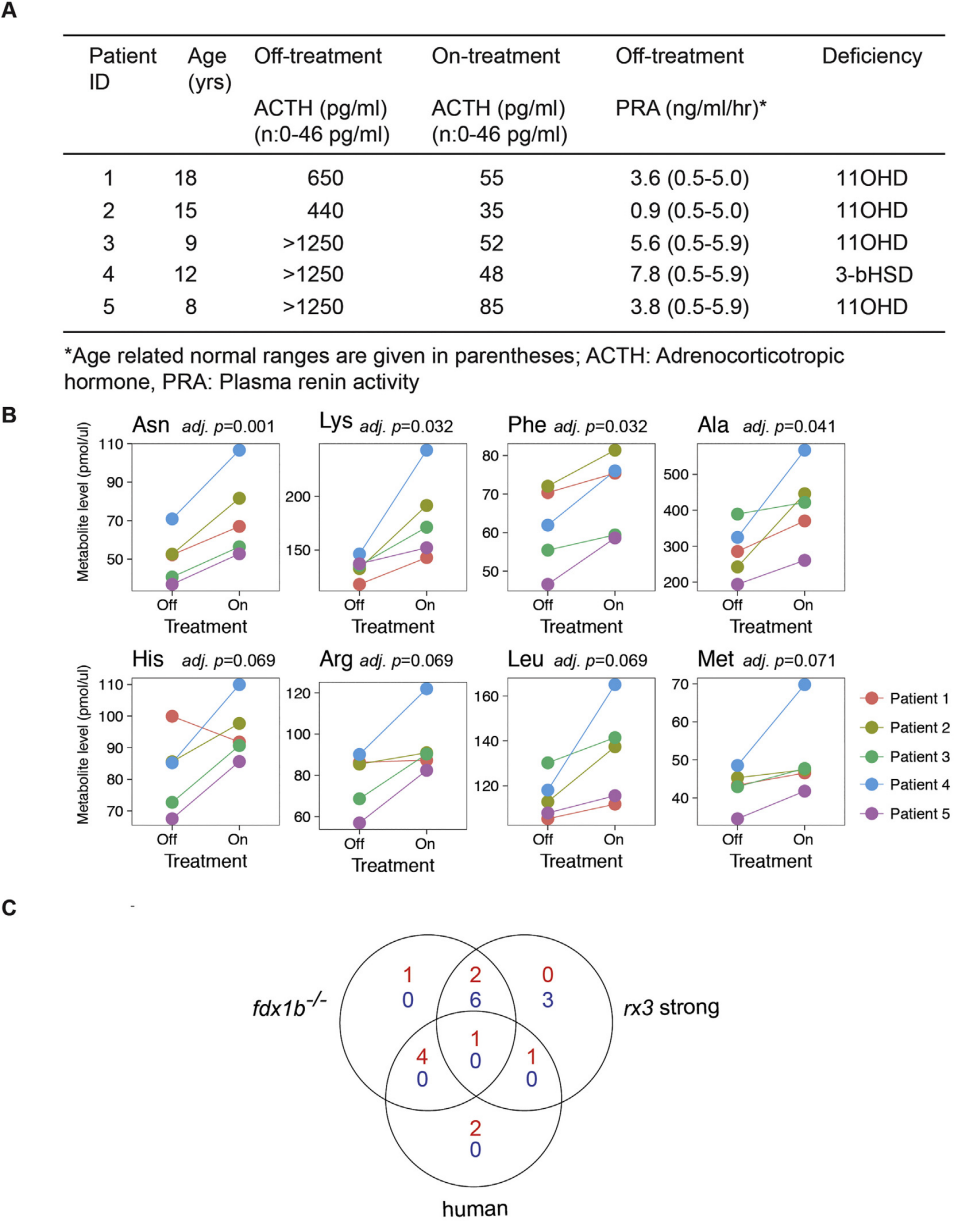
Amino acid changes in plasma of patients with primary adrenal insufficiency overlap with changes observed in *fdx1b*^*−/−*^ mutant larvae. (A) Hormonal features of patients with primary adrenal insufficiency on hydrocortisone treatment and after 48 h interruption of therapy. (B) Levels of the amino acids asparagine (Asn), lysine (Lys), phenylalanine (Phe), alanine (Ala), histidine (His), arginine (Arg), leucine (Leu), and methionine (Met) in human patient plasma in On and Off treatment conditions. (C) Venn diagram showing the altered amino acids in human patients with primary insufficiency, *fdx1b*^*−/−*^ and *rx3* strong mutants between untreated and treated conditions. More amino acids are altered between human and *fdx1b*^*−/−*^ mutants (*p* = .02; assessed by hypergeometric testing) than between human and *rx3* strong mutants (*p* = .18). The data for the *rx3* mutants is a reanalysis from our previously published data set [74].

### 3.7 *fdx1b*^*−/−*^ mutants exhibit systemic changes distinct from a secondary adrenal insufficiency-like phenotype

To explore if metabolic changes observed in *fdx1b* mutant larvae are solely explained by the lack of GCs, we compared the results obtained from the *fdx1* larvae to *rx3* strong mutant larvae, another model for GC deficiency. Therefore, we reanalysed our recently published *rx3* strong mutants RNA-seq data set ([74]; Fig. S5 and Table S6). *fdx1b*- mutant larvae present a phenotype similar to primary adrenal insufficiency in humans, including cortisol deficiency, down-regulation of GC-dependent marker genes, an up-regulation of the HPI axis [[33]; Fig. 6A] and interrenal hyperplasia (Fig. 6B). In contrast, the *rx3* strong mutant larvae show a profoundly impaired synthesis of GCs due a reduction in ACTH-producing corticotrope cells [[16]; Fig. 6A]. Indeed, ACTH target genes are mainly up-regulated in *fdx1b*^*−/−*^ mutants (p = .008) and down-regulated in *rx3* strong mutants (p = .006) (Fig. 6C). These findings confirm the assumption that *fdx1b*-deficient larvae resemble a primary adrenal insufficiency phenotype and that *rx3* strong mutants can be used to model secondary adrenal insufficiency. Thus, we subsequently explored if these two zebrafish models of adrenal insufficiency differ on a global gene expression level. A statistical model including an interaction term between the mutant type and the phenotype was employed to extract the differences between gene expression changes in the *fdx1b*^*−/−*^ and *rx3* strong models compared to their respective controls. The next step assessed which GO term and KEGG-based metabolic gene sets were enriched in the differentially expressed genes with a significant interaction term. Pathways such as the ornithine urea cycle and branched-chain amino acids show the same changes in both mutants (see [74] for the *rx3* strong and Fig. S6 for the *fdx1b*^*−/−*^ mutants). However, several metabolic pathways showed enrichment for differences between *fdx1b*^*−/−*^ and *rx3* strong mutants (Fig. 6D and E). These pathways include sterol biosynthesis and steroid hormone biosynthesis (Fig. 6D–F). Consequent with the detected changes in ACTH levels, steroid hormone biosynthesis genes are up-regulated in *fdx1b*^*−/−*^ and down-regulated in *rx3* strong mutants (Fig. 6E and F).

Furthermore, glutathione metabolism was identified as a pathway with major alterations (Fig. 6D and E). Interestingly, GC-deficient *fdx1b*^*−/−*^ mutants show an overall up-regulation (*p* = 1.52E-11), whereas *rx3* strong mutants show no clear global up- or down-regulation of glutathione metabolism-associated genes despite having the same level of GC deficiency as the *fdx1b*^*−/−*^ larvae. Consistent with differential redox metabolism changes in the two mutants, Nrf2 target genes were mainly down-regulated in *rx3* strong mutants (Fig. S3A and Table S6), in contrast to the *fdx1b*^*−/−*^ situation (Fig. S3A). This further supports the conclusion reached above that the observed higher oxidative stress levels in *fdx1b*^*−/−*^ mutants are only partially caused by the lack of GCs. Importantly, several other metabolic pathways, including tryptophan metabolism; valine, leucine and isoleucine degradation; purine metabolism; alanine, aspartate and glutamate metabolism; and terpenoid backbone synthesis differ between both mutants (Fig. 6D and E). Overall, our data show profound global differences in the two types of GC deficiency models. Thus, such pathophysiological differences in models of GC deficiency might most probably be caused by dysregulation of other pathways and factors associated with the specific cause of impaired GC synthesis rather than only cortisol deficiency.

### 3.8 Patients with adrenal insufficiency show similar amino acid changes as *fdx1b*^*−/−*^ zebrafish mutants

To explore if humans with primary adrenal insufficiency have similar metabolic changes as observed in *fdx1b*^*−/−*^ mutants, we performed targeted metabolic profiling of amino acids on blood samples from children with primary GC deficiency (Fig. 7A). Off treatment, patients showed changes in asparagine, lysine, phenylalanine, alanine, histidine, arginine, leucine and methionine concentrations (Fig. 7B). These data indicate that amino acid metabolism is strongly affected when GC replacement is insufficient. Interestingly, alanine, arginine, leucine, methionine and phenylalanine concentrations are also lower in untreated *fdx1b*^*−/−*^ larvae compared to DEX-treated *fdx1b*^*−/−*^ larvae (Fig. 7C and Table S4). A smaller similarity was observed when comparing the data to *rx3* strong mutants. Histidine and methionine were the only two out of the eight amino acids altered in patients, being similarly affected in *rx3* strong mutants (Fig. 7C and Fig. S7). Thus overall, the metabolic profiles in patients with primary adrenal insufficiency correlate better with the metabolic profile observed in *fdx1b*^*−/−*^ mutants than with that of *rx3* strong mutants. This further shows that there are differences between both types of GC deficiency models and that the *fdx1b*^*−/−*^ mutant recapitulates more closely metabolic changes of patients with primary adrenal insufficiency.

## 4 Discussion

By combining transcriptomics and metabolic profiling, we detected reprogramming of metabolism, such as glutamine metabolism in GC-deficient *fdx1b*^*−/−*^ mutant larvae. In line with this observation, glutamine concentrations were increased in *fdx1b*^*−/−*^ mutant larvae. Due to the relevance of glutamine for several linked metabolic pathways [13] which were also affected in *fdx1b*^*−/−*^ mutants, we further characterised these pathways in the zebrafish model. Notably, we provide the first spatio-temporal characterization of the zebrafish glutaminase and glutamine synthetase genes at embryonic/larval stages, whereas some data on the synthetases are available in adult zebrafish [14]. Thus, our analysis provides a valuable source for future investigation into the role of glutamine in cancer development and growth [2,13] using the zebrafish model [51,80]. Importantly, *gls2a* and *gls2b* were the only differentially expressed genes in glutaminolysis or synthesis in *fdx1b*^*−/−*^ mutants, and were also responsive to GC treatment. This finding supports the GC dependent regulation of the diurnal transcription of *gls2a* [74], and the relevance of regulation of glutaminolysis by GCs. The GC dependent regulation of *gls2a* and *gls2b*, and the fact that zebrafish are not self-feeding at the examined stages, strongly suggests that increased glutamine levels in *fdx1b*^*−/−*^ mutant larvae are likely to be caused by impaired glutaminolysis rather than by impaired glutamine synthesis.

Importantly, data on global metabolic changes in patients with adrenal insufficiency are limited. Our clinical data obtained from patients with primary adrenal insufficiency indicate metabolic alterations similar to observed changes in our *fdx1b*-deficient larval model. However, plasma of untreated patients does not show the major accumulation of glutamine detected in *fdx1b*^*−/−*^ mutants. As glutamine metabolism involves several organs [73], the difference may be due to the larger set of tissues analysed in the whole larval extracts, but could also reflect species-specific differences in, for example, nitrogenic waste excretion [72]. Nevertheless, eight other amino acids showed lower plasma concentrations in untreated patients, and five of these were also lower in untreated *fdx1b*^*−/−*^ mutants compared to DEX treatment. Furthermore, two of these amino acids were also changed in plasma samples of congenital adrenal hyperplasia (CAH) patients under GC replacement therapy [3]. Asparagine and methionine were lower in patients with CAH treated with lower GC doses than in those receiving high doses of GC. Furthermore, the purine inosine was increased in patients receiving low dose GC treatment [3]. This correlates with the observed metabolic and broad transcriptional as well as post-transcriptional up-regulation of purine metabolism observed in untreated *fdx1b*^*−/−*^ mutants. Of note, the metabolic alterations in patients with primary adrenal insufficiency show greater overlap with those seen in the *fdx1b*^*−/−*^ mutant than with those in *rx3* mutants. This observation indicates that such changes may be specific to primary adrenal insufficiency as opposed to conditions associated with secondary adrenal insufficiency. Overall, by determining metabolic alterations most likely specific to primary adrenal insufficiency, our study expands the understanding into metabolic changes in patients with primary GC deficiency. Furthermore, overlapping alterations in larvae validate *fdx1b*^*−/−*^ mutant as a model for metabolic features of primary adrenal insufficiency.

To explore potential changes between primary and secondary adrenal insufficiency, we compared our results from the *fdx1b*^*−/−*^ mutant with our previously published data set of *rx3* strong mutants [74], which represents a model of secondary adrenal insufficiency. This analysis showed clear differences in changed gene expression in the two systems consistent with the different causes of GC deficiency. Down-regulation of ACTH target gene expression and steroidogenic genes expression in the ACTH-deficient *rx3* strong model, in contrast to the up-regulation observed in *fdx1b*^*−/−*^ mutants, support the notion that these different lines represent models of secondary and primary adrenal insufficiency, respectively. Furthermore, we found striking differences in the metabolic profiles between the *rx3* strong and *fdx1b*^*−/−*^ mutants. This includes glutathione metabolism, which was the most affected pathway in *fdx1b*^*−/−*^ mutants. The observed changes in glutathione metabolism suggest altered levels of oxidative stress, which have been implicated in pathogenesis by leading to metabolic dysfunction [46] or DNA lesions [69]. The up-regulation of *Gst* genes and Nrf2 target genes, as well as changes in GSH/GSSG ratio and taurine concentrations provide further evidence of increased oxidative stress in *fdx1b*^*−/−*^ larvae.

The altered oxidative stress response in *fdx1b*^*−/−*^ mutants is expected to be caused by the lack of GCs. This appears surprising in light of observations that GC excess increases reactive oxygen production and suppresses the Nrf2 mediated antioxidant response by silencing Nrf2 target genes [1,43]. These effects result in increased oxidative stress and have been suggested to be a key mechanism for the adverse effect of GCs. The increased transcript levels of the Nrf2 target gene *fth1* in the *fdx1b*^*−/−*^ mutant larvae were restored by GC replacement suggesting a regulatory role of GCs in the oxidative stress response in zebrafish larvae. However, the detected changes in GSH/GSSG ratio and taurine levels were only partially restored by GC replacement. Furthermore, the vast majority of Nrf2 target genes and genes of glutathione metabolism showed opposing transcriptional patterns between the GC-deficient *fdx1b*^*−/−*^ and *rx3* strong mutants, and thus the altered oxidative stress levels in *fdx1b*^*−/−*^ mutants cannot solely be explained by the lack of GCs. Given that steroid hormone synthesis is one of the main contributors to the production of reactive oxygen species in mitochondria [65], it is tempting to speculate that the detected increase in oxidative stress in the *fdx1b*^*−/−*^ mutant is a consequence of an increased electron leakage due to insufficient Fdx1b-mediated electron transfer during steroidogenesis. This assumption is supported by reports in rodents and human showing that defects in nicotinamide nucleotide transhydrogenase (*NNT*), a gene that is involved in the maintenance of the mitochondrial redox homeostasis, leads to disturbances in adrenal redox status and increased levels of oxidative stress [55,56,78]. Overall, it appears that GCs modulate the antioxidant response, but GC deficiency might not directly lead to oxidative stress.

Although some evidence suggests GC-dependent post-transcriptional gene regulation in immune system and anti-inflammatory pathways [70], the wider understanding of post-transcriptional regulation of metabolic pathways by GCs remains elusive. Our study identified *de novo* purine synthesis as a metabolic pathway affected in *fdx1b*^*−/−*^ mutants. We demonstrated the GC-dependent post-transcriptional regulation of two key enzymes in this pathway, *paics* and *atic*. The pharmacological inhibition of Gls revealed a correlation between regulation of *paics* with changes in glutamine or glutamine-linked pathways. This is in line with previous observations that glutamine can induce *paics* expression in human lung cancer cell lines [31]. However, glutamine levels *per se* seem not to induce *paics* mRNA levels, as YAP transgenic zebrafish which exhibit also increased glutamine levels [12] show repressed *paics* mRNA levels. Why is *paics* expression differentially affected in the two conditions of glutamine accumulation? The first committed and rate-liming entry of glutamine into *de novo* purine biosynthesis is catalysed by *phosphoribosyl pyrophosphate amidotransferase* (*ppat*) [62]. Expression of this enzyme positively correlates with *paics* mRNA levels in YAP transgenic (*p* = .0502) and *fdx1b*^*−/−*^ mutants (*p* = .0006). Thus, lower expression of *ppat* in YAP transgenics may limit glutamine flux into the purine biosynthesis pathway, while glutamine flux would be increased in *fdx1b*^*−/−*^ mutants. This observation suggests that the flux of glutamine into *de novo* purine synthesis rather than absolute glutamine concentrations are most likely linked to *paics* mRNA stability.

In contrast, post-transcriptional regulation of *atic* appears to be glutamine-independent. miRNAs are key molecules for post-transcriptional regulation [34], and are promising candidates in the regulation of *paics* and *atic*. Indeed, several miRNAs have been shown to regulate glutamine metabolism through targeting glutaminase expression [27,52,66] and may thereby affect the purine biosynthesis pathway genes. In our study, we have identified the miRNA *dre-mir-2192* as being differentially expressed in *fdx1b*^*−/−*^ mutants. Importantly, *dre-mir-2192* has been predicted to target metabolism in fish species [39]. A murine deletion model of DICER shows only a slight decrease in both transcription and mRNA abundance of *paics.* The high correlation of mRNA and pre-mRNA levels supports the assumption that the observed post-transcriptional changes of *paics* in *fdx1b*^*−/−*^ mutants are driven by a miRNA-independent mechanism. *atic* shows an opposing profile as mRNA levels drop to a higher extent in DICER KO mice than those of pre-mRNA, suggesting that miRNAs are involved in the post-transcriptional stabilization of *atic* mRNA. However, a potential direct regulation of *atic* by *dre-mir-2192* would be rather surprising, as *dre-mir-2192* would have to act through a yet undescribed mechanism stabilizing the transcript rather than targeting it for degradation. Alternatively, the regulation of *atic* by miRNAs might be indirect, for example, involving the destabilization of another regulator of *atic*, which then allows for stabilization of *atic* transcript levels. Such regulators inducing mRNA decay have been described in the context of GCs [15,61] and the nutrient sensing mTOR pathway [47]. Intriguingly, *dre-mir-2192* shares a seed match with *atic* but not *paics* at the 3’UTR [48]. Future work will be required to understand whether and how miRNAs regulate the post-transcriptional regulation of *atic*. Overall, our identification of GC mediated post-transcriptional regulation of the *de novo* purine synthesis pathway defines new research questions aiming at a detailed mechanistic understanding of the interconnections between GC signalling, glutamine metabolism and miRNA regulation.

In conclusion, our work provides a detailed characterization of the *in vivo* consequences of a disrupted ferredoxin system and GC deficiency on metabolism at a transcriptional, post-transcriptional and metabolite level. Based on our characterization of two GC-deficient mutants, we propose that the *fdx1b*^*−/−*^ and *rx3* strong mutant have translational capacity for future investigations into the pathogenesis of primary and secondary adrenal insufficiency. Given that zebrafish is a well-known model organism for performing high-throughput screenings, future applications of both models might include screenings to identify compounds that specifically target the metabolic differences between patients with primary and secondary adrenal insufficiency.

The following are the supplementary data related to this article.

Table S1 Protein sequences for the generation of the phylogenetic tree Table S1

Table S2 qRT-PCR primers used for the transcriptional analysis Table S2

Table S3 PCR oligo sequences used for the generation of the whole-mount *in situ* probes Table S3

Table S4 Metabolic profiling results and stats Table S4

Table S5 RNA-Seq data *fdx1b*^*−/−*^*vs.* siblings Table S5

Table S6 RNA-seq data *rx3* strong *vs.* weak Table S6

Table S7 Gene set enrichment analysis based on the curated gene sets (C2) from MSigDB Table S7

Supplementary material Image 1
