# Supplementary material for: Glucocorticoid deficiency causes transcriptional and post-transcriptional reprogramming of glutamine metabolism
Source: eBioMedicine. 2018 Sep 26;36:376–89. doi: 10.1016/j.ebiom.2018.09.024 (PMC6197330; doi:10.1016/j.ebiom.2018.09.024)
Supplement: Supplementary file 8 — Supplementary material [file mmc8.docx]

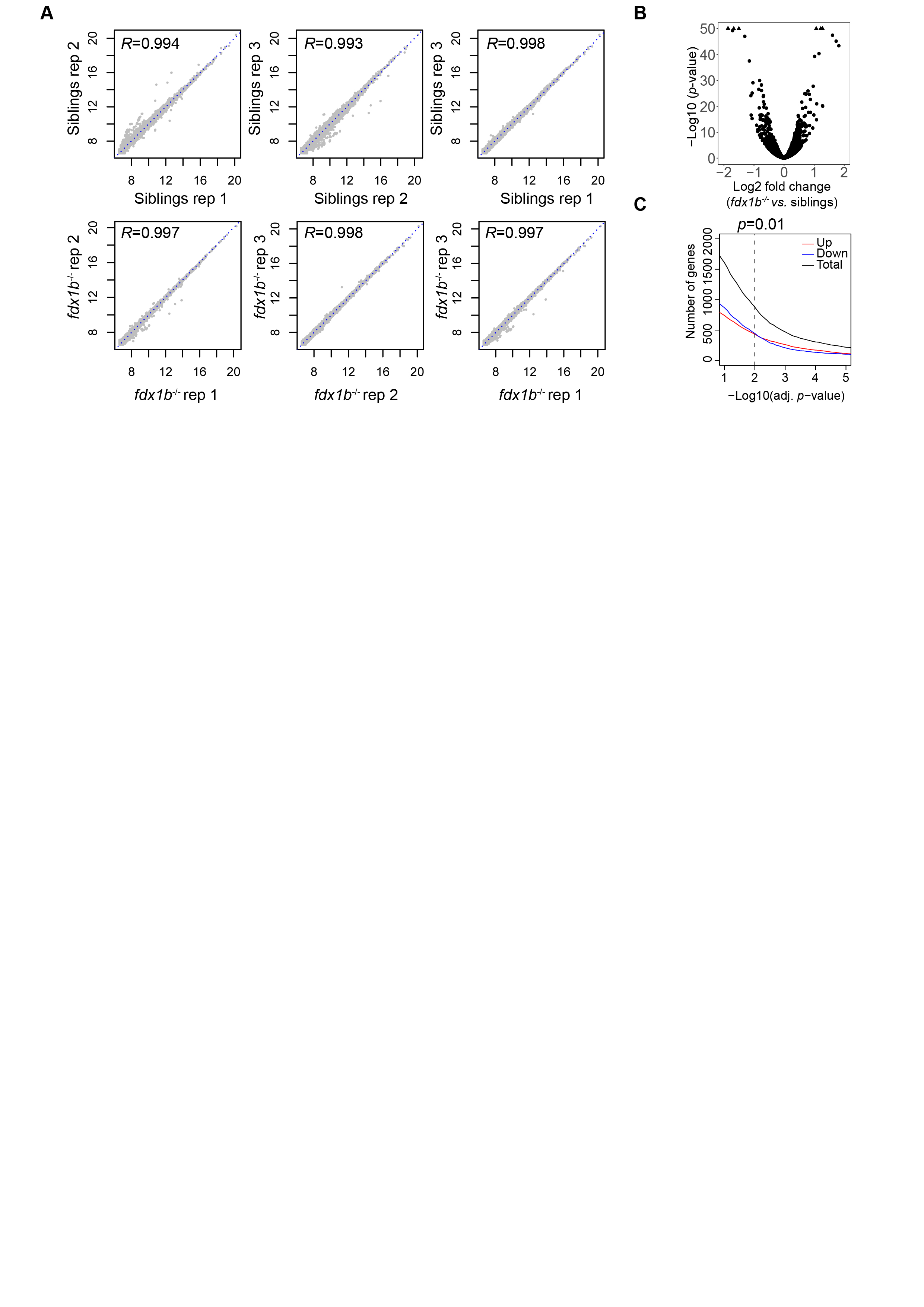


**Figure S1. Quality assessment of the *fdx1b^-/-^* larval RNA-seq study**

(A) Reproducibility of biological replicates (rep) of *fdx1b^-/-^* mutant and wild-type larval samples. Log2 of total normalized counts. *R* indicates Pearson correlation at the top of each panel. (B) Volcano plot of differences in mRNA expression between *fdx1b^-/-^* and wild-type siblings. (C) Number of differentially expressed genes in *fdx1b^-/-^* mutant larvae plotted against the adjusted *p*-value. Up-regulated genes in red; downregulated genes in blue; all differentially expressed genes in black.


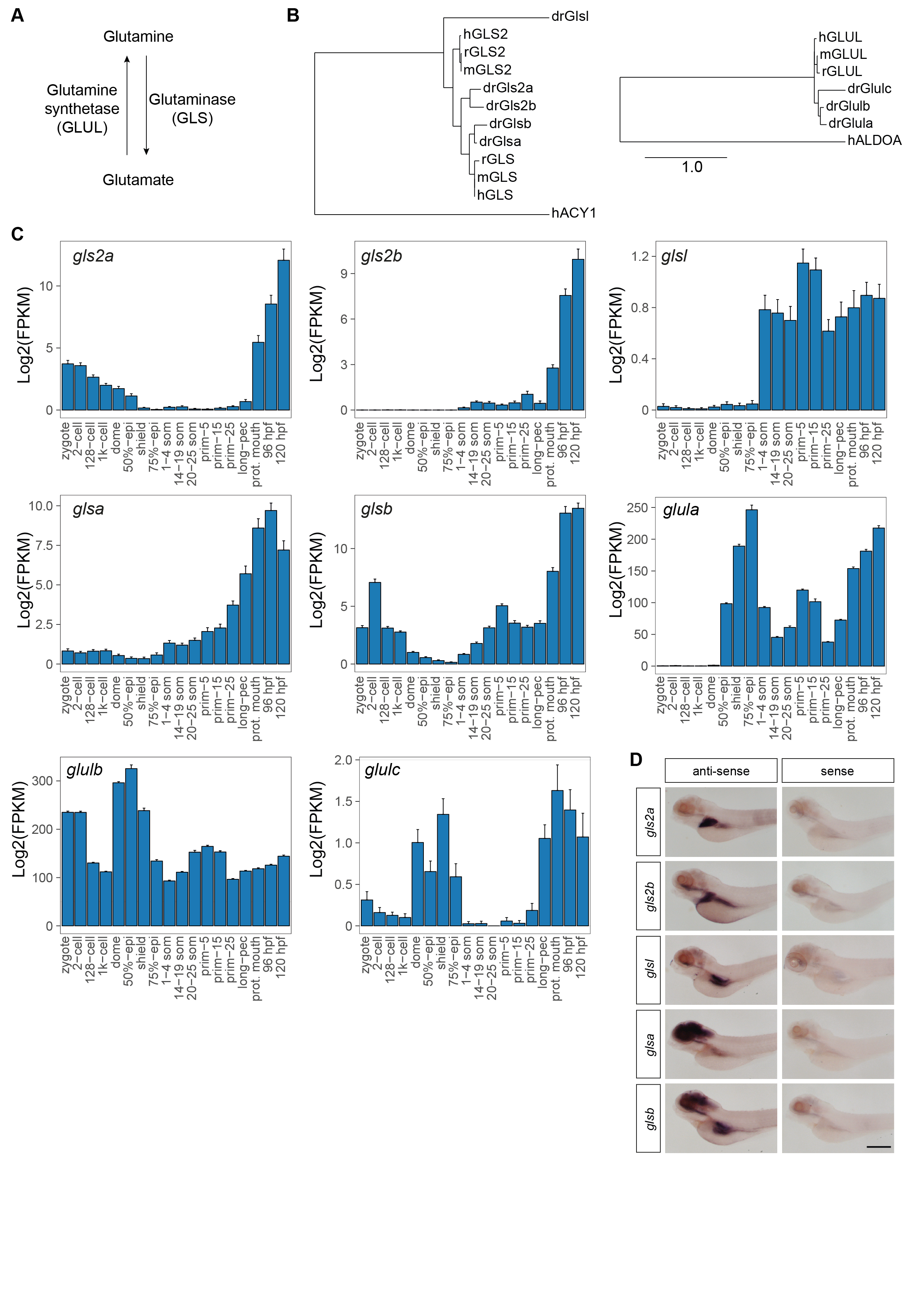


**Figure S2. Glutamine metabolism in vertebrates.**

(A) Schematic illustrates glutamate metabolism in humans. (B) Phylogenetic analysis of the glutamine synthetase (GLUL) and glutaminase (GLS, GLS2) proteins in human (h), mouse (m), rat (r) and zebrafish (dr). Aminoacylase 1 (ACY1) and aldolase A (ALDOA) were used as outgroups. (C) Reanalysis of a previously published RNA-seq study (1) showing expression of the zebrafish glutaminases (*gls2a*, *gls2b*, *gls3, glsa, glsb*) and glutamine synthetase genes (*glula, glulb, glulc*) in wild-type embryos/larvae during development. Abbreviations: epi, epiboly; som, somites; prim, primordium; prot, protruding; hpf, hours post-fertilization. (D) Whole-mount *in situ* hybridization of the glutaminase transcripts (*gls2a*, *gls2b*, *gls3*, *glsa*, *glsb*) in wild-type larvae at 120 hpf. Images are a representative example of the whole-mount *in situ* hybridization stainings (n = 20). Scale-Bar: 0.2 mm.


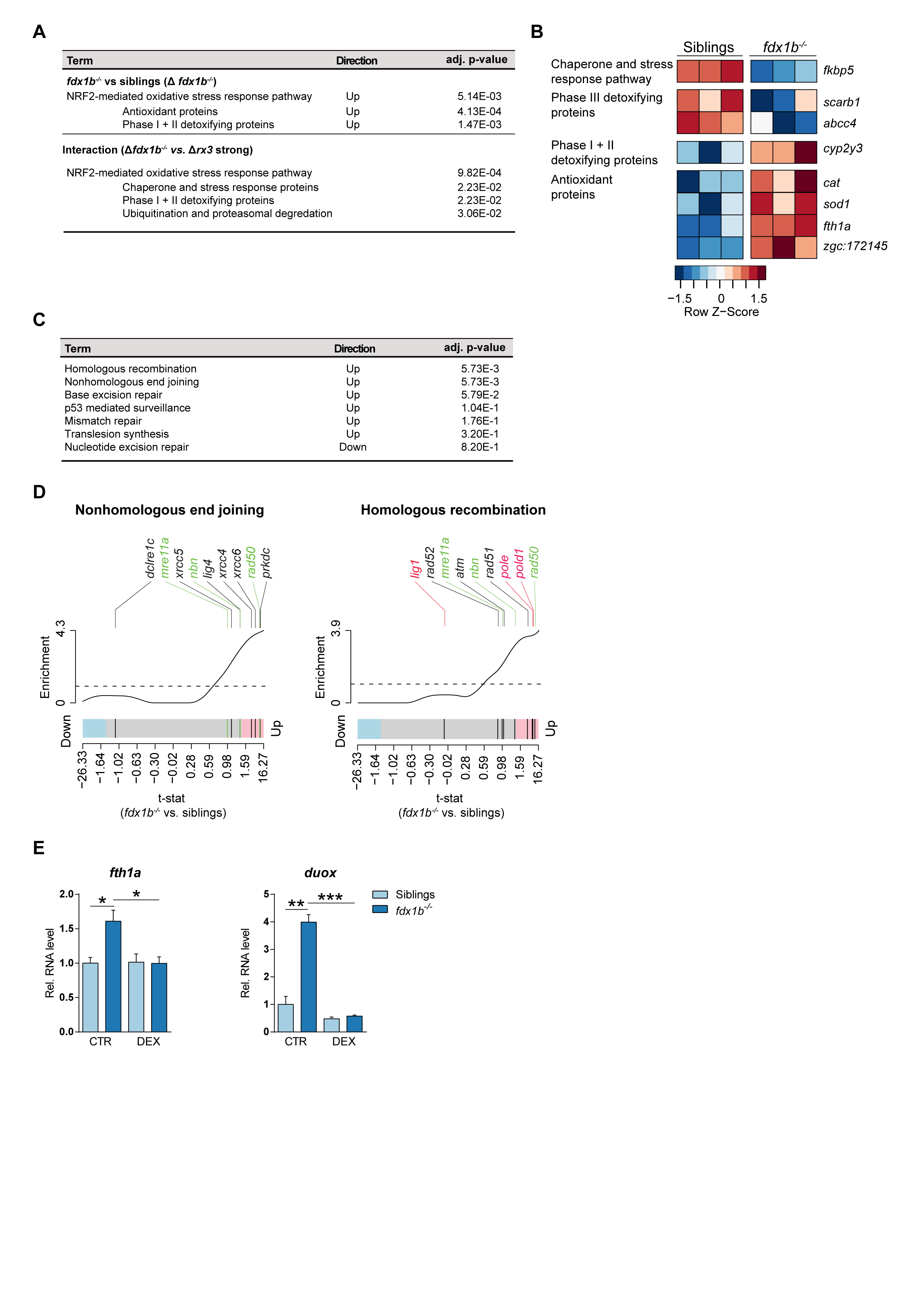


**Figure S3. Transcriptional analysis of Nrf2 and DNA repair pathways in *fdx1b^-/-^* mutants.**

(A) Gene-set enrichment of differentially expressed oxidative stress-responsive genes. (B) Heatmap represents the differentially expressed Nrf2-mediated oxidative stress-responsive genes in *fdx1b^-/-^* mutant larvae. The genes are grouped according to their functional category. (C) Gene-set enrichment of differentially expressed DNA repair genes. (D) Barcode plots for DNA repair pathways in *fdx1b^-/-^* mutant larvae in comparison to the wild-type siblings. In green labelled are genes shared between nonhomologous end joining and homologous recombination and in red labelled are genes shared between homologous recombination and base excision repair. (E) qRT-PCR analysis of *fth1* and *duox* in *fdx1b^-/-^* mutant larvae and wild-type siblings (120 hpf) in the absence (CTR) or presence of DEX.


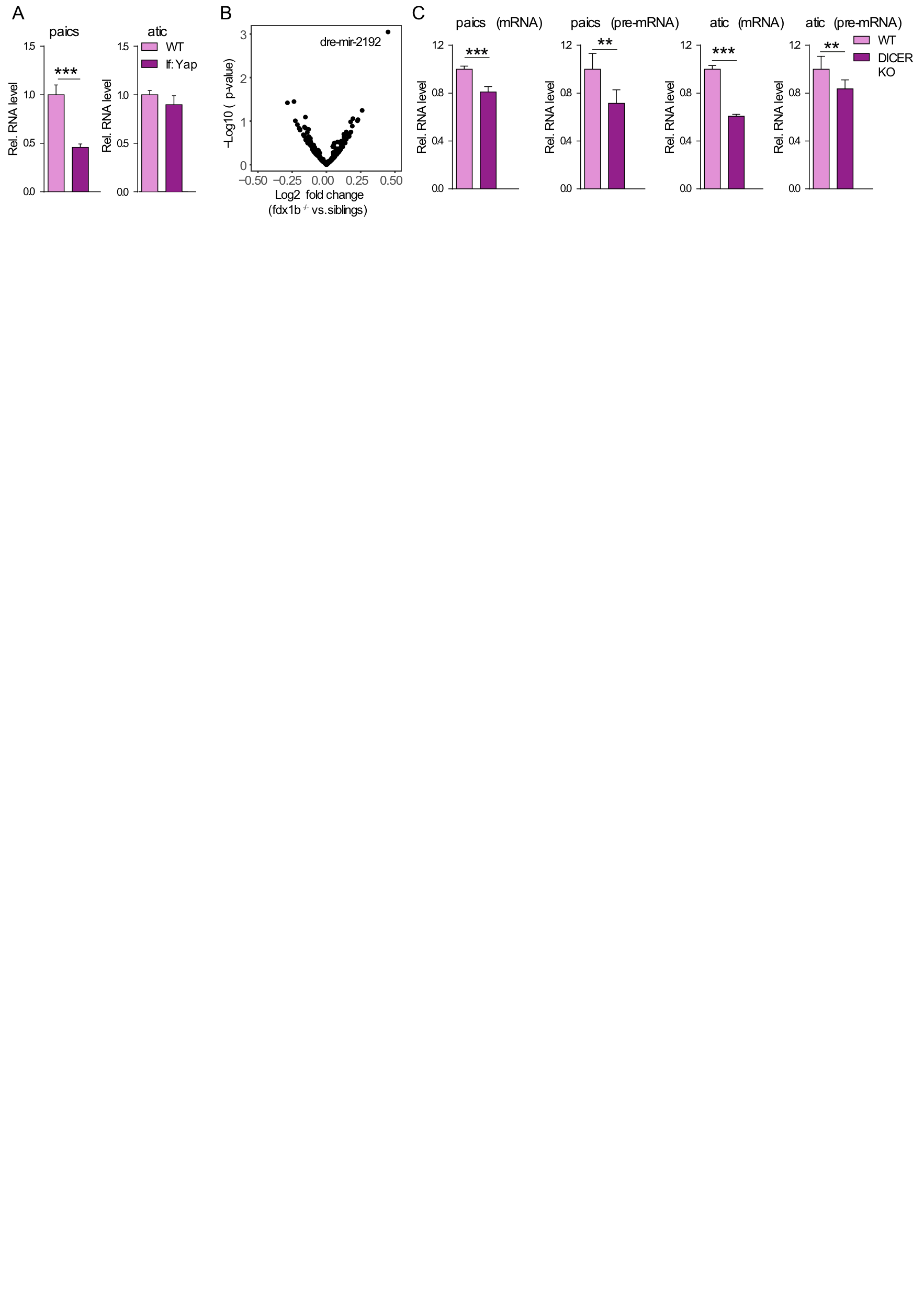
**Figure S4. Potential mechanisms of post-transcriptional regulation of *paics* and *atic* by glucocorticoids.**

(A) mRNA levels of *paics* and *atic* in a zebrafish model of reprogrammed glutamine metabolism (*lf:YAP*) (2). (B) Volcano plot of differences in detected miRNA expression between *fdx1b^-/-^* and wild-type siblings. (C) mRNA and pre-mRNA levels of *paics* and *atic* in a mouse model of liver-specific inactivation of miRNA biogenesis (DICER knockouts (KOs)) (3). Interaction [genotype:region(intron/exon)]: *paics* (*p* = 0.54) and *atic* (*p* = 0.03).


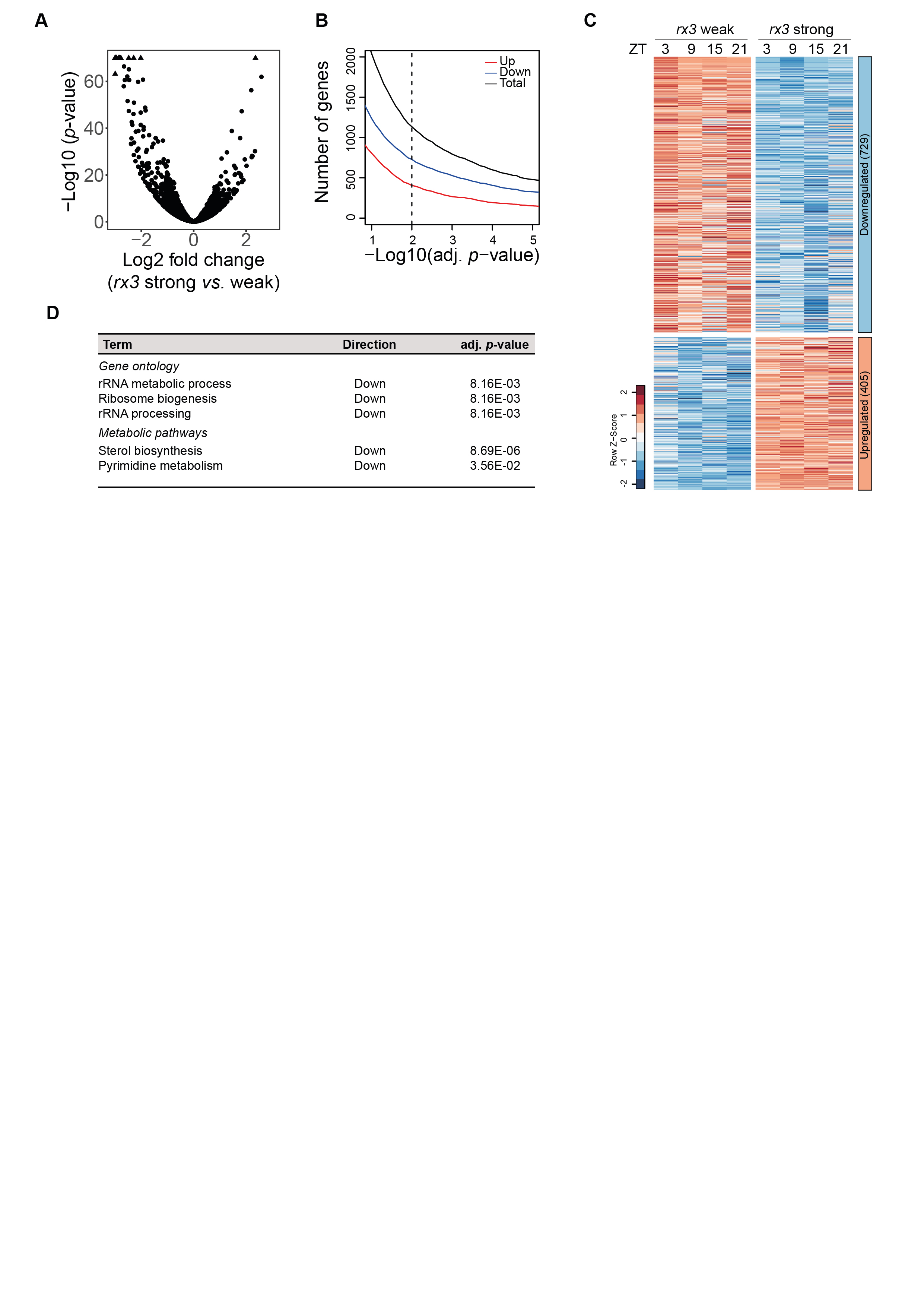
**Figure S5. Differential gene expression in *rx3* larvae as examined by RNA-seq.**

(A) Volcano plot of differences in mRNA expression between *rx3 strong* and *weak* mutant larvae. (B) Number of differentially expressed genes in function of the adjusted *p*-value. (C) Heatmaps of normalized mRNA expression levels of genes in *rx3 weak* and *strong* mutant larvae and wild-type siblings. Red, high expression; blue, low expression. (D) Gene enrichment analysis of differentially expressed genes (*rx3* strong *vs* *rx3* weak larvae). The data for the *rx3* mutants is a reanalysis from our previously published dataset (4).


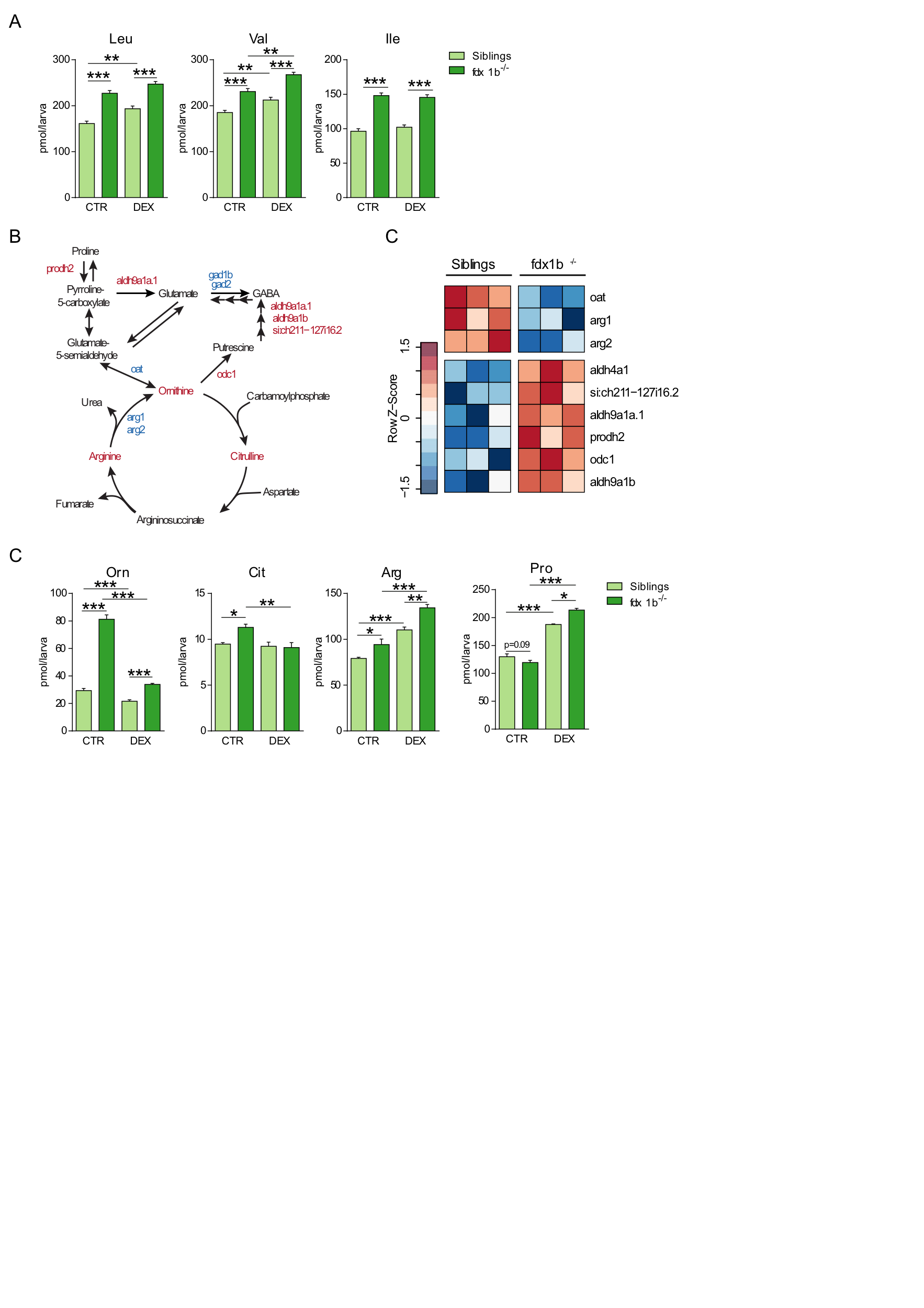


**Figure S6. *fdx1b^-/-^* mutant larvae exhibit alterations in valine, leucine, and isoleucine degradation, and in the ornithine-urea cycle.**

(A) Metabolite levels of leucine (Leu), valine (Val) and isoleucine (Ile) in *fdx1b^-/-^* mutant and wild-type sibling larvae (120 hpf) in the absence [vehicle as a control (CTR)] or presence of dexamethasone (DEX). (B) Schematic illustrates the ornithine-urea cycle. Metabolites and genes of this pathway altered in *fdx1b^-/-^* larvae compared to wild-type siblings are marked in red for up-regulation and blue for down-regulation. (C) Heatmap showing differentially expressed genes of the ornithine-urea cycle in *fdx1b^-/-^* mutant larvae as analysed by RNA-seq. (D) UPLC-FLR based analysis of ornithine (Orn), citrulline (Cit), arginine (Arg) and proline (Pro) in *fdx1b^-/-^* mutant and wild-type sibling larvae (120 hpf) in the absence [vehicle as a control (CTR)] or presence of dexamethasone (DEX).


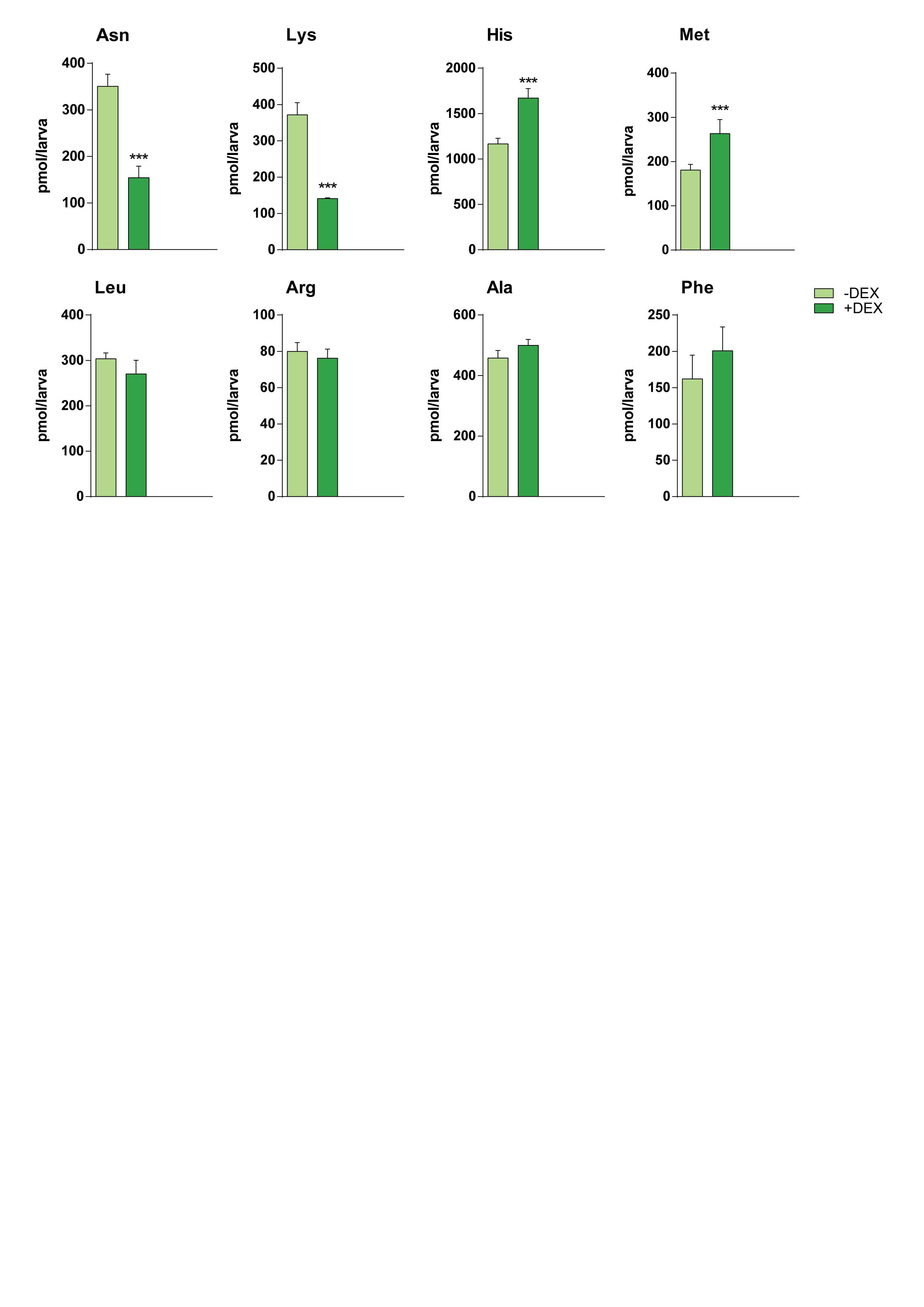


**Figure S7. Amino acid level changes in *rx3* strong mutants.**

Bar diagram shows level of methionine (Met), arginine (Arg), alanine (Ala), phenylalanine (Phe), leucine (Leu), histidine (His), asparagine (Asn) and lysine (Lys) between dexamethasone treated (+DEX) and untreated (-DEX)*rx3* strong mutants. *, *p* < 0.05, **, *p* < 0.01, ***, *p*< 0.001.

**Table S1. Protein sequences for the generation of the phylogenetic tree**

**Table S2. qRT-PCR primers used for the transcriptional analysis**

**Table S3. PCR oligo sequences used for the generation of the whole-mount *in situ* probes**

**Table S4. Metabolic profiling results and stats**

**Table S5. RNA-Seq data *fdx1b^-/-^*** ***vs.* siblings**

**Table S6. RNA-seq data *rx3* strong *vs.* weak**

**Table S7. Gene set enrichment analysis based on the curated gene sets (C2) from MSigDB**

**References**

1. White RJ, et al. (2017) A high-resolution mRNA expression time course of embryonic development in zebrafish. *bioRxiv*.

2. Cox AG, et al. (2016) Yap reprograms glutamine metabolism to increase nucleotide biosynthesis and enable liver growth. *Nat Cell Biol* 18(8):886–896.

3. Du N-H, Arpat AB, De Matos M, Gatfield D (2014) MicroRNAs shape circadian hepatic gene expression on a transcriptome-wide scale. *Elife* 3:e02510.

4. Weger BD, et al. (2016) Extensive Regulation of Diurnal Transcription and Metabolism by Glucocorticoids. *PLoS Genet* 12(12):e1006512.
